# Evaluation of Genetic or Cellular Impairments in Type I IFN Immunity in a Cohort of Young Adults with Critical COVID-19

**DOI:** 10.1007/s10875-023-01641-1

**Published:** 2024-01-17

**Authors:** L. E. Covill, A. Sendel, T. M. Campbell, I. Piiroinen, S. Lind Enoksson, E. Wahren Borgström, S. Hansen, K. Ma, P. Marits, A. C. Norlin, C. I. E. Smith, J. Kåhlin, L. I. Eriksson, P. Bergman, Y. T. Bryceson

**Affiliations:** 1 Center for Hematology and Regenerative Medicine, Department of Medicine, Karolinska Institute, Stockholm, Sweden; 2 Division of Clinical Immunology and Transfusion Medicine, Karolinska University Hospital, Stockholm, Sweden; 3 Division of Infectious Diseases, Karolinska University Hospital, Stockholm, Sweden; 4 Department of Laboratory Medicine, Karolinska Institutet, Stockholm, Sweden; 5 Division of Perioperative Medicine and Intensive Care, Karolinska University Hospital, Stockholm, Sweden; 6 Broegelmann Laboratory, Department of Clinical Sciences, University of Bergen, Bergen, Norway

**Keywords:** SARS-CoV-2, Primary immunodeficiency, Inborn error of immunity, Type 1 interferon signaling

## Abstract

Several genetic and immunological risk factors for severe COVID-19 have been identified, with monogenic conditions relating to 13 genes of type I interferon (IFN) immunity proposed to explain 4.8% of critical cases. However, previous cohorts have been clinically heterogeneous and were not subjected to thorough genetic and immunological analyses. We therefore aimed to systematically investigate the prevalence of rare genetic variants causing inborn errors of immunity (IEI) and functionally interrogate the type I IFN pathway in young adults that suffered from critical COVID-19 yet lacked comorbidities. We selected and clinically characterized a cohort of 38 previously healthy individuals under 50 years of age who were treated in intensive care units due to critical COVID-19. Blood samples were collected after convalescence. Two patients had IFN-α autoantibodies. Genome sequencing revealed very rare variants in the type I IFN pathway in 31.6% of the patients, which was similar to controls. Analyses of cryopreserved leukocytes did not indicate any defect in plasmacytoid dendritic cell sensing of TLR7 and TLR9 agonists in patients carrying variants in these pathways. However, lymphocyte STAT phosphorylation and protein upregulation upon IFN-α stimulation revealed three possible cases of impaired type I IFN signaling in carriers of rare variants. Together, our results suggest a strategy of functional screening followed by genome analyses and biochemical validation to uncover undiagnosed causes of critical COVID-19.

**Supplementary Information:**

The online version contains supplementary material available at 10.1007/s10875-023-01641-1.

## Introduction

In December 2019, cases of flu-like disease were first reported resulting from infection with a novel coronavirus, SARS-CoV-2 [1, 2]. As of 2023, cumulative incidence of the resultant disease, COVID-19 [3], has surpassed 500 million cases, with deaths exceeding 6 million [4]. Disease severity ranges from asymptomatic to death [5–7]; complications include cytokine storm, respiratory failure with acute respiratory distress syndrome (ARDS) [8], and multi-organ failure [9–13]. COVID-19 presents with a wide range of less debilitating clinical characteristics, the most common being cough, myalgia, headache, and fever [8, 9, 14]. During the first pandemic wave, the infection fatality rate was estimated to 0.15–0.82% [15–17]. Several risk factors have been identified for severe illness, including age above 50 years, male sex, and comorbidities such as obesity, hypertension, diabetes, kidney disease, and severe asthma [18–27].

Multiple independent efforts have been made to identify genetic contributors to critical COVID-19. Genome-wide association studies (GWAS) performed across patient groups with different levels of disease severity [28, 29] found that the strongest association with severe disease is conferred by a risk locus on chr3p21.31. The highest linkage disequilibrium is at an intronic polymorphism (rs17713054) in *LZTFL1* [30], encoding a protein involved in cilia function [31–33]. Further associations are with the ABO blood group locus and in interferon (IFN) type I-related loci including *IFNAR1* and *TYK2*, and IFN-induced genes *OAS1*, *OAS2*, and *OAS3* [28, 29]. Functional assessments exploring mechanisms by which these variants confer risk allow the possibility of personalized therapy development targeting those affected. However, the utility of these data for clinical risk prediction has not been evaluated. Furthermore, interpreting results of these studies faces the challenge of applying risk loci from ethnically homogenous cohorts to ICU patients of more varied ethnic backgrounds.

Monogenic diseases have also been uncovered in COVID-19 patients. Zhang et al. studied a global cohort of patients with critical COVID-19 (defined as requiring ventilation, developing septic shock, or organ failure), reporting enrichment of rare, predicted loss-of-function (LoF) variants in 13 genes associated with susceptibility to influenza A pneumonia with roles in viral sensing and type I IFN signaling [34]. They concluded that genetic deficiencies in these signaling pathways may dramatically increase the risk of critical COVID-19, accounting for up to 4.8% of such cases [34–37], or up to 10.7% in children under the age of 16 who suffered critical COVID-19 [38]. Specifically, X-linked LoF variants in *TLR7*, encoding a receptor for single-stranded RNA highly expressed in plasmacytoid dendritic cells (pDCs), have been estimated to cause 1% of critical COVID-19 cases in males under 60 years old [39–41]. Furthermore, patients were identified with rare biallelic LoF variants in autosomal recessive genes previously associated with susceptibility to severe influenza virus infection, including variants in *TLR3*, *IRF7*, *IRF9*, *IFNAR1*, and *IFNAR2* [42–46]. Only rare missense and predicted LoF variants were considered in these analyses, potentially underestimating the burden of non-coding variants reducing expression of crucial gene loci [47]. Furthermore, although the effect of these biallelic or hemizygous variants on disease has been established in patient cells and in vitro assays, the disease contribution of heterozygous variants in autosomal recessive genes remains unclear. Notably, a genetic meta-analysis by Povysil et al. identified only a single LoF variant in the 13 loci examined by Zhang et al. out of 713 severe COVID-19 cases [48]. Povysil et al. observed that since missense variants in controls were not characterized, a direct comparison of cases and controls in Zhang et al. was not possible. We therefore hypothesized that functional assessment of relevant pathways in patients may aid the diagnosis of inborn errors of immunity (IEI) in critical COVID-19 patients by identifying individuals with, e.g*.*, heterozygous dominant negative or non-coding LoF variants.

Although many IEI patients diagnosed before their infection with SARS-CoV-2 required hospitalization, those who died usually had further risk factors [49–51]. However, IEIs associated with autoantibodies to type I IFN (hereafter referred to as autoantibodies), e.g., *AIRE* variants causing autoimmune polyglandular syndrome type 1 (APS-1), significantly increased the risk of life-threatening disease after SARS-CoV-2 infection [52–54]. Autoantibodies have been identified in 5.2–10.0% of severe cases where no IEI is diagnosed, with increases in prevalence commensurate to age [55–61]. Further studies can validate these figures and determine if these assays are useful for directing targeted treatment in the clinic.

We assembled a cohort of adults with critical COVID-19 treated at the Karolinska University Hospital intensive care units (ICU). To reduce other risk factors which may confound any genetic contribution to disease, we limited our study to patients under 50 years of age, who lacked comorbidities, such as cancer or known causes of secondary immunodeficiency. We performed comprehensive clinical, immunological, and genetic analyses of these critical COVID-19 patients during convalescence to evaluate the efficacy of functional assays and potentially identify new variants causative of IEI.

## Methods

### Cohort Collection

The study was conducted in accordance with ethical application Dnr 2020–01911, approved by the Swedish Ethical Review Board. Informed written consent was obtained from all patients (*n* = 38) and samples were collected between October 2020–November 2021 and processed accordingly. For cellular analyses, donors from the Karolinska Hospital Blood Bank represented healthy controls.

### Intensive Care Unit Data Collection

Data on physiological parameters in the ICU, severity scoring, information on invasive ventilation, comorbidities, and laboratory parameters was extracted from the ICU medical record systems Clinisoft and Take Care.

### Genetic Analyses

Genomic DNA was extracted from whole blood. Whole-genome sequencing was performed as previously described [62]. Base GWAS data was retrieved from the GENOMICC summary statistics depository r3 [29]. PRSice-2 was used to calculate PRS on our cohort and the 1kGP [63]. R packages ggplot2 and pROC were used to create density plots and receiving operator characteristic (ROC) curves. R package logistf was used in gene and pathway burden testing as specified in the supplemental methods.

### Immunological Analyses

Absolute cell numbers and phenotype were quantified in whole blood. For functional assays, peripheral blood mononuclear cells (PBMCs) from cryopreserved samples were stimulated as detailed in the supplemental methods. Patient plasma was screened for autoantibodies.

### Statistical Testing of Functional Assays

Statistical analyses were performed with R (version 4.1.1).

## Results

### Demographic and Clinical Characterization of Critical COVID-19 Patient Cohort

**Fig. 1:**
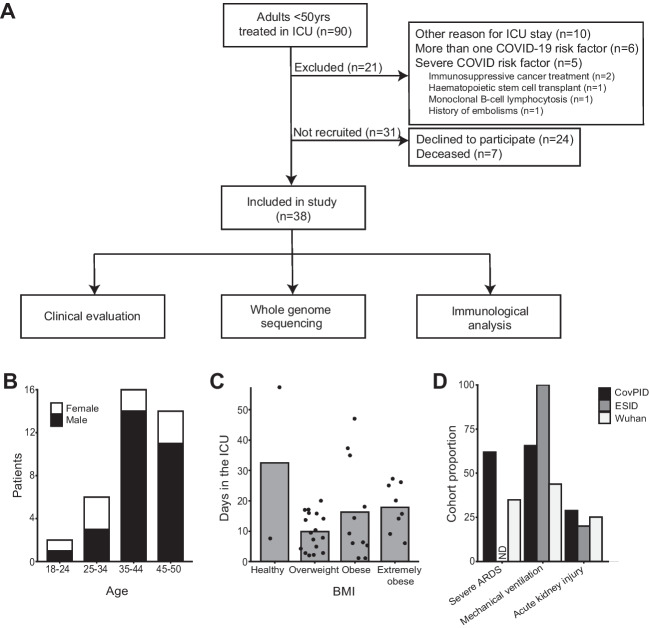
Patient demographics in the CovPID-20 cohort. **A** Flowchart detailing the exclusion, invitation, and acceptance of patients to join the cohort for further clinical, functional, and genetic analysis. **B** Distribution of cohort patients over sex and age range; black bars represent males. **C** Plot depicts the BMI of each patient against their total time spent in the ICU. **D** Proportion of patients who had each complication of critical COVID-19 in our cohort; the ESID cohort studied by Meyts et al. and the Wuhan cohort studied by Yu et al. complications indicating disease severity were severe ARDS, defined as degree of hypoxemia (Pa_O2_/Fi_O2_ ratio) ≤ 100 [64]; mechanical ventilation required; and acute kidney injury

We identified patients aged 18–50 years treated in the ICU in the Karolinska University Hospital, during March 2020–March 2021 with SARS-CoV-2 infection (Fig. 1A). Of 90 patients, seven were deceased and samples unobtainable. Twenty-one were excluded for having other reasons for admission to the ICU, medical conditions associated with severe immunosuppression (i.e., stem cell transplantation, immunosuppressive treatments, or cancer), or more than one COVID-19-related risk factor (diabetes, obesity, asthma, kidney disease, and hypertension). Sixty-three individuals meeting the study criteria were invited to a follow-up clinical visit with optional study participation (Fig. 1A). Twenty-five patients declined to participate. Of 38 patients who enrolled, 76.3% were male and the average age was 40.2 ± 7.8 (Table 1, Fig. 1B). Average BMI was 31.9 ± 6.4 kg/m^2^ (Fig. 1C, Supplementary Fig. 1A). Regarding COVID-19 risk factors, included were 3 patients with diabetes; 1 with kidney disease; 8 with asthma; and 4 with hypertension. We reasoned that our strict exclusion criteria could increase the likelihood of identifying individuals with IEI.

**Table 1:** Cohort demographics and clinical characteristics

Demographic and clinical data		*n* = 38	%	Range	SD
	Age (at ICU admission, years, mean)	40.1		18.2–49.8	7.6
	Sex – male	29	76.3%		
	Sex – female	9	23.7%		
Comorbidities					
	BMI (at ICU admission, mean)	31.9		23.4–51	6.3
	Diabetes mellitus type 2	4	10.5%		
	Hypertension	4	10.5%		
	Asthma	8	21.1%		
	CKD stage 2	1	2.6%		
	Vitamin D (nmol/L, mean)	41.8		18–98	19.3
Ethnicity					
	Middle Eastern	14	36.8%		
	East African	5	13.2%		
	Northern European	4	10.5%		
	Southeast Asian	4	10.5%		
	Southeast European	4	10.5%		
	South American	2	5.3%		
	Central American	1	2.6%		
	Eastern European	1	2.6%		
	Western Asian	1	2.6%		
	Unknown	2	5.3%		
ICU clinical data					
	Length of ICU stay (days, mean)	14.6		1–57	12.5
	Days between ICU admission and assessment (days, mean)	249.2		166–344	48.5
	Invasive ventilation	25	65.8%		
	Tracheostomy	12	31.6%		
	ECMO	3	7.9%		
	Prone positioning	2	60.5%		
	Acute kidney injury	4	1%		
	Continuous renal replacement therapy	6	15.8%		
	Pulmonary flow index, (P_a_O2/F_i_O2; kPa)	12.6		6–28	4.8
	*Mild ARDS (PFI* > *26.6 kPa)*	*1*	*2.6%*		
	*Moderate ARDS (PFI 13.3–26.6 kPa)*	*13*	*34.2%*		
	*Severe ARDS (PFI* < *13.3 kPa)*	*24*	*63.2%*		
	SAPS III score	46.9		32–59	7.2
	SOFA score (max)	5.8		2–13	3.5
	SOFA score (mean)	4.5		2–9.1	2.4

Self-assessed symptom scoring was collected retroactively for the time of admission, 3 months after discharge, and time of follow-up (Supplementary Fig. 1B), demonstrating a typical constellation of symptoms during disease and convalescence for critical COVID-19 [8]. The progression of disease in our cohort was similar to subsets of critical COVID-19 patients in two other clinically characterized groups of patients: a multi-center study of patients admitted to Wuhan ICUs in February 2020 (“Wuhan cohort,” *n* = 226) [65]; and patients with pre-diagnosed IEIs who were followed during SARS-CoV-2 infection (“ESID cohort,” *n* = 15) [51] (Fig. 1D). Thus, despite our selection criteria of relative youth and lack of comorbidities, our cohort displayed objective features of critical COVID-19 disease with single to multiple organ failure requiring intensive care.

### Immune Phenotypes of the COVID-19 Patient Cohort

Lymphocyte subpopulation numbers and frequencies were analyzed on blood samples drawn at the time of follow-up (mean = 249 days post-ICU discharge). Relative to adult healthy controls [66], a small increase was observed in frequencies of central memory CD4^+^ T cells (*p* = 0.04, unpaired *T*-test), although no change was seen in central memory CD8^+^ T cells nor in activation markers CD38^+^ or HLA-DR^+^ [66], suggesting that cells were not activated above baseline (Supplementary Fig. 2A).

Lymphocyte reactivity was assessed by flow cytometric assay for specific cell-mediated immune responses in activated whole blood (FASCIA) on samples drawn at the time of follow-up [67]. Six patients (15.8%) showed a decreased reactivity of CD4^+^ T cells to SARS-CoV-2 whole virus compared to 65 seropositive healthy controls, whereas four (10.5%) patients showed a markedly increased reactivity (Supplementary Fig. 2B). Increased reactivity was interpreted as prolonged immune activation and decreased reactivity may reflect an impaired immune response or memory formation.

### Anti-type I Interferon Autoantibody Analysis of the COVID-19 Patient Cohort

**Fig. 2:**
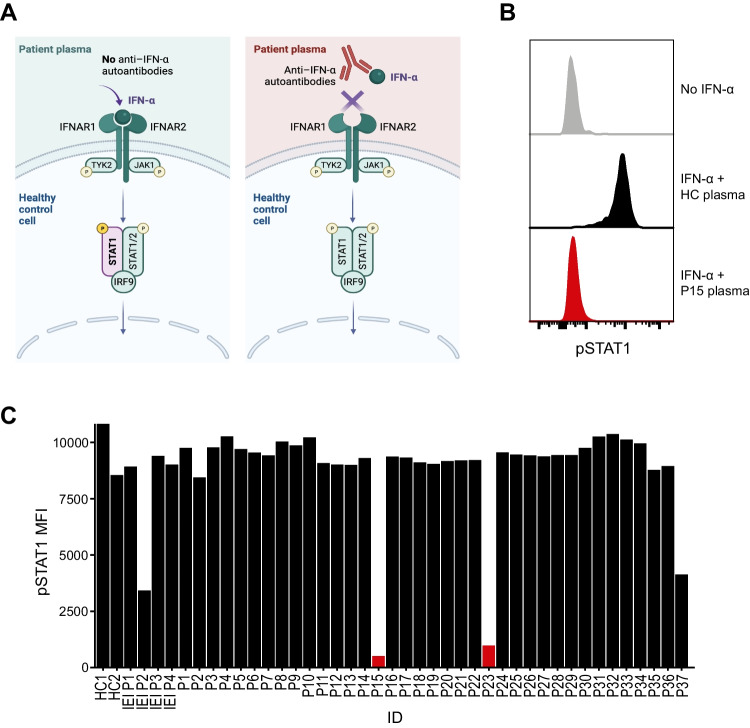
Screening of patient plasma for autoantibodies to type I IFN. **A** Diagram depicts the anti-IFN-α autoantibody detection assay. IFN-α stimulation in the presence of patient plasma without anti-IFN-α autoantibodies (left panel) signals through the type I IFN receptor and is detected through increased phosphorylation of STAT1 (purple). Patient plasma containing anti-IFN-α autoantibodies neutralize IFN-α (right panel), resulting in diminished STAT1 phosphorylation. **B** Example histogram shows pSTAT1 levels in monocytes when unstimulated (gray), stimulated with IFN-α in the presence of healthy control (HC) plasma (black), and when stimulated in the presence of serum from a patient with autoantibodies (P15; red). **C** Measurement of pSTAT1 MFI when stimulated with IFN-α in the presence of plasma from healthy controls (HC), previously diagnosed patients with IEI in IRF7 (IEI P1 and P2) or TLR7 (IEI P3 and P4), and the COVID-19 patient cohort (P1-37). Patients with detectable anti-IFN-α autoantibodies are indicated in red

Autoantibodies to type I IFN constitute a biological risk factor for critical COVID-19, but in the general population are only present in 0.18–0.30% of individuals below 70 years of age [55, 68]. To identify whether any patients in our cohort harbored autoantibodies, we assessed plasma from patients during convalescence. Patient plasma samples were mixed with recombinant human IFN-α2 and incubated with healthy donor PBMCs. Fixed cells were stained with anti-phosphorylated STAT1 (pSTAT1) and assessed by flow cytometry (Fig. 2A). Of the 38 patients, two had neutralizing autoantibodies to IFN-α2, comprising 5.3% of patients in our cohort (Fig. 2B, C). As such, severe COVID-19 in two of the 38 patients could be explained by acquired IFN type I autoantibodies. Notably, one of the patients identified with autoantibodies, P15 (who also had the longest ICU stay of 57 days), was later diagnosed with a thymoma.

### Common Genetic Variant Contribution to Critical COVID-19

**Fig. 3:**
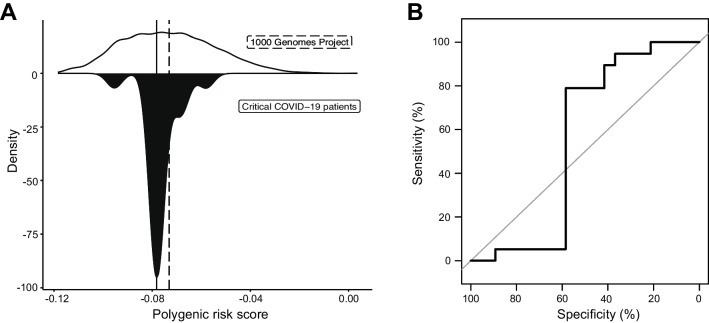
Analysis of polygenic risk score association with critical COVID-19. **A** Density plot of the PRS calculated for the 1kG cohort (upper) and the COVID-19 patient cohort (lower). **B** ROC curve showing the sensitivity and specificity of PRS in separating the two groups, as indicated

To identify genetic factors predisposing to disease, all patients were subjected to whole-genome sequencing. GWAS have previously identified the locus with the greatest risk score for severe COVID-19 as rs17713054G > A [28], corresponding to a gain-of-function (GoF) variant in *LZTFL1* [56]. We observed a 4.0-fold enrichment of rs17713054 in a heterozygous state in our cohort (MAF = 0.224 compared to MAF = 0.055 in GnomAD; *p* = 0.0000084, X-squared test) suggesting that common risk variants could be explanatory in our cohort. We also calculated an odds ratio (OR) adjusted for ancestry principal components (PCs) of 5.23 (CI = 2.51–10.76; *p* = 0.000018) when our cohort was compared to the 1000 Genomes Project (1kGP). Summary statistics from a study by GENOMICC were used to perform polygenic risk score (PRS) analysis to calculate the degree to which common COVID-19 risk variants were predisposing our patients to severe disease [29]. Using the 1kGP as a control group, the mean PRS in case and control groups was determined (Fig. 3A), giving an area under the curve (AUC) of 0.552 (Fig. 3B). These results indicate that common risk variants only partially explain disease severity in our cohort. The separation between our cohort and control individuals was not significant. Thus, PRS did not prove a useful predictive tool in young and previously healthy critical COVID-19 patients.

### Analysis of Very Rare Genetic Variants in Type I IFN Pathways

**Fig. 4:**
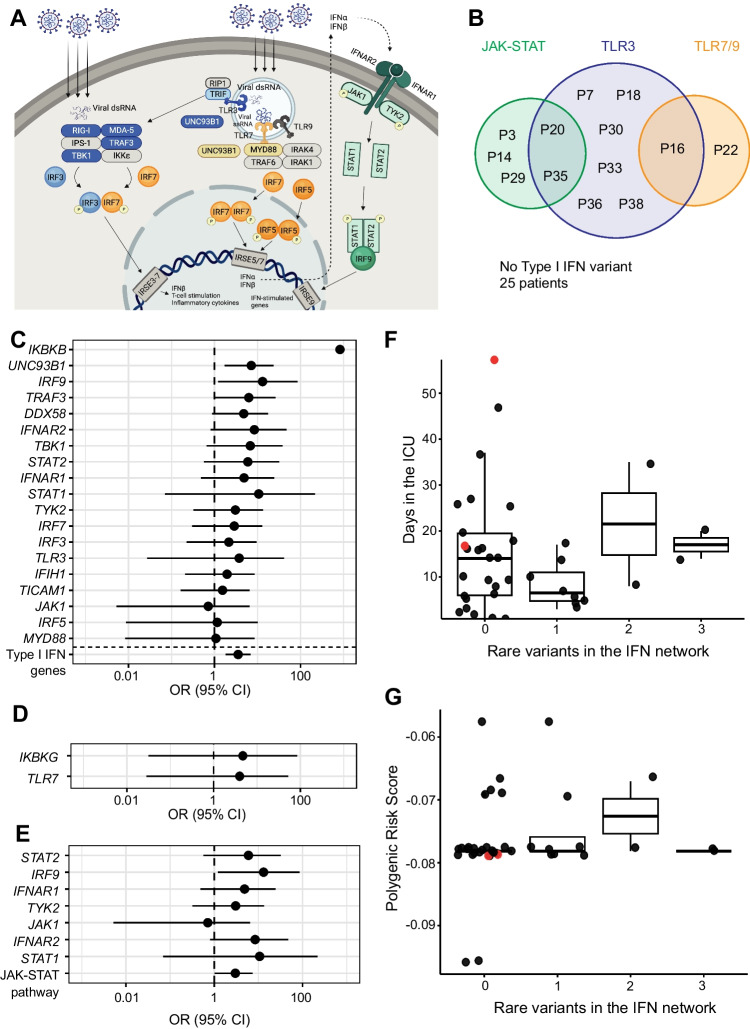
Identification of very rare variants in type I IFN pathway genes. **A** Plot depicts the primary known proteins involved in type I IFN production and signaling, divided into three visually distinct pathways. Proteins coded for by genes included in the study are colored by pathway: TLR3-stimulated IFN production is in blue, TLR7/TLR9-stimulated IFN production is in orange, and IFN signaling via the type I IFN pathway is in green. These type I IFN proteins were collated from field literature and are evaluated in subsequent analysis. Proteins visualized in gray were not included in the genetic analyses. **B** Venn diagram of cohort patients carrying variants with very rare GnomAD MAF < 0.001 variants in any one or more of the three pathways defined in **A**. **C** Forest plot of odds ratios (95% confidence intervals) of very rare variants above the 99% mutation significance cutoff in autosomal genes involved in type I IFN production and signaling; and the sum of very rare variants in individual genes in the type I IFN signaling pathway; compared to very rare variants meeting the same pathogenicity prediction criteria in individuals in the 1kGP dataset. Ancestry PCs were calculated for patients in the CovPID-20 cohort and individuals in 1kGP and the first four were included in odds ratio analysis as covariates. **D** Forest plot of odds ratios (95% confidence intervals) of very rare variants above the 99% mutation significance cutoff in X-linked genes involved in type I IFN production identified in male patients, compared to very rare variants meeting the same pathogenicity prediction criteria in male individuals from the 1kGP dataset. **E** Boxplots show the distribution of time spent in the ICU for patients with 0–3 rare variants with MAF < 0.001 in the type I IFN pathway. No correlation was observed between variant carriership and time required to recover from critical COVID-19. Patients with anti-IFN autoantibodies detected are represented in red. **F** Boxplots show PRS distribution for individuals with 0–3 identified genetic variants with MAF < 0.001 in the type I IFN pathway. Patients with anti-IFN autoantibodies detected are represented in red

In previous studies of familial clusters of patients with severe COVID-19 across Sweden, we identified two brothers with a homozygous LoF *IRF7* variant [43], as well as two brothers with a novel X-linked *TLR7* LoF variant [39]. Although these patients did not meet the current study criteria, we hypothesized that similar monogenic afflictions may explain additional sporadic cases of critical COVID-19 in young, formerly healthy individuals. We analyzed very rare variants (MAF < 0.001 in GnomAD) in 21 gene loci involved in type I IFN production and signaling in all study patients (Fig. 4A, Supplementary Table 2 [69–74]). Very rare variants were identified in 31.6% of the patients (Table 2, Fig. 4B), after filtering by predicted pathogenicity by including only variants with CADD score over a gene-specific tolerance threshold calculated by the mutation significance cutoff (MSC) set at 99% [75, 76]. However, such variants were also present in 21.2% of the 1kGP cohort (*p* = 0.12, unpaired *T*-test). No structural variants in any of the genes were identified in the patients (Supplementary Fig. 3A-S). To understand the impact of all type I IFN pathways on disease, OR analysis was performed on all patients and controls for autosomal genes (Fig. 4C) and on male patients and controls for X-linked genes (Fig. 4D). Significant enrichment of rare, predicted damaging variants was observed in *IKBKB*, *UNC93B1*, *IRF9*, and *TRAF3*, but a clear overall enrichment of variants across type I IFN genes was not apparent. Ethnicity was used as a covariate when constructing the model (Supplementary Fig. 4A). The 1kGP was used as a control cohort to calculate PCs in ancestry analysis, and for OR analysis. To determine the validity of significant results amongst the type I IFN genes, this analysis was repeated with a gene set associated with handedness, i.e., not linked to viral immunity, and each with the approximate metrics of a type I IFN gene included in the analysis. Overall, genes associated with type I IFN and with handedness demonstrated similar distributions of statistically significant enrichment (Supplementary Fig. 5A), suggesting that there may be an inflation in OR caused by small numbers of observations. However, the OR of a summary of the variants in all autosomal genes considered displayed statistical enrichment in type I IFN genes (*p* = 0.0003) whilst handedness genes did not (*p* = 0.09). Moreover, since rare variants were identified in five pathway components, we also evaluated enrichment of rare variants in the type I IFN signaling pathway in our cohort compared to the 1kGP and found significant enrichment (*p* = 0.045, Table 3, Fig. 4E), which was absent in the overall assessment of the counterpart handedness genes (*p* > 0.05, Supplementary Fig. 5B). The absence of clear enrichment across type I IFN genes compared to handedness genes suggested that increasing the number of genes evaluated is unlikely to substantially increase diagnostic yield, even in young and previously healthy COVID-19 patients.

**Table 2:** Coding and splice region variants with MAF less than 0.001 identified in type I IFN-associated genes in the cohort of 38 ICU patients

Individual	Ethnicity	Gene	Reference transcript	Nucleotide change	Amino acid change	Genotype	Inheritance model	Global MAF	Population MAF	CADD score
P3	Kurdish	*IRF9*	ENST00000396864	c.991 + 232G > C	Splice variant	Heterozygous	AR	0.0001124	-	6.1
P7	Kurdish	*IRF3*	ENST00000601291	c.1252G > A	p.Ala418Thr	Heterozygous	AD	0.0007794	-	15.2
P14	Central American	*TYK2*	ENST00000525621	c.1153C > T	p.His385Tyr	Heterozygous	AR	0	-	26.1
P16	Kenyan	*UNC93B1*	ENST00000227471	c.610A > C	p.Met204Leu	Heterozygous		0.000008038	0.0001301	10.1
				c.1774G > C	p.Gly592Arg	Heterozygous^a^		0.0003254	0.001093	17.4
				c.1778C > T	p.Pro593Leu	Heterozygous^a^		0	0	15.7
P20	Somalian	*STAT2*	ENST00000557235	c.1020A > G	splice variant	Heterozygous	AR	0.00003981	0	14.5
		*IKBKB*	ENST00000519735		p.Trp254Ter	Heterozygous	AR; AD	0.0008	0,0002	2.6
P22	Syrian	*IRF7*	ENST00000397574	c.1122C > T	Splice variant	Heterozygous	AR	0.00004726	-	12.4
P26	South Korean	*IKBKG*	ENST00000369601	c.549G > C	p.Gln183His	Heterozygous	XR, XD	0	0	22.3
P29	Kurdish	*IFNAR2*	ENST00000342136	c.884C > T	p.Pro295Leu	Heterozygous	AR	0.00008954	-	9.3
P30	Turkish	*TRAF3*	ENST00000560371	c.1099G > A	p.Val367Met	Heterozygous		0.00008495	-	22.2
P33	Unknown	*TBK1*	ENST00000331710	c.965A > G	p.His322Arg	Heterozygous	AD	0.000008356	-	23.6
P35	Armenian	*IFNAR1*	ENST00000270139	c.308G > A	p.Arg103His	Heterozygous	AR	0.00006016	-	26.2
		*TICAM1*	ENST00000248244	c.784G > A	p.Ala262Thr	Heterozygous	AR; AD	0.000004128	-	8.9
		*IFIH1*	ENST00000263642	c.2962G > T	p.Val988Leu	Heterozygous	AR; AD	0.0004128	-	25.4
P36	Filipino	*DDX58*	ENST00000379883	c.1991G > A	p.Arg664His	Heterozygous^a^	AD	0.00005007	0.0004888	23.6
				c.2014 + 8 T > C	Splice variant	Heterozygous^a^		0.0001628	0.0004924	7.7
P38	Albanian	*TRAF3*	ENST00000560371	c.-18 + 4A > C	Splice variant	Heterozygous		0	0	10.3

^a^Occurs on the same haplotype with another variant

**Table 3:** Carriers of very rare variants above the 99% MSC threshold, in autosomal IFN genes

	Carriers in cases			Carriers in controls		
Gene	Heterozygous	Homozygous	Possible compound heterozygous	Heterozygous	Homozygous	Possible compound heterozygous
*DDX58*	1	0	0	51	0	0
*IFIH1*	1	0	0	35	0	2
*IFNAR1*	1	0	0	28	0	0
*IFNAR2*	1	0	0	21	0	0
*IKBKB*	1	0	0	1	0	0
*IRF3*	1	0	0	43	0	0
*IRF5*	0	0	0	25	0	0
*IRF7*	1	0	0	44	0	1
*IRF9*	1	0	0	17	0	0
*JAK1*	0	0	0	36	0	0
*MYD88*	0	0	0	28	0	0
*STAT1*	0	0	0	20	0	0
*STAT2*	1	0	0	10	0	1
*TBK1*	1	0	0	35	0	0
*TICAM1*	1	0	0	83	0	1
*TLR3*	0	0	0	11	0	0
*TRAF3*	2	0	0	13	0	0
*TYK2*	1	0	0	30	0	1
*UNC93B1*	0	0	1	61	0	4
All included genes	10	0	2	517	0	10

We also noted that some patients were carriers of more than one heterozygous variant within one or more pathways under investigation. Such individuals could experience a cumulative oligogenic effect of functional differences in two or three proteins, as has been previously observed in IEI [77–79]. Thus, we assessed the impact of variant carriership on duration and severity of illness. However, increased carriership of variants in these pathways did not correlate with length of time spent in the ICU (Fig. 4E) or with any of the parameters used in the ICU to measure disease severity (Table 1). Furthermore, no significant association was observed between variant carriership and obesity, which could have suggested that very rare IFN gene variants were increasing the risk of individuals with healthy BMI and no risk factors.

Rare variants may convey missing heritability from GWAS data, which includes common variants only [80–83]. We therefore hypothesized that taken together, PRS and rare variants in the IFN pathways may give a better prediction of COVID-19 severity than either variable individually. Initially, correlation between rare variants and PRS was evaluated and found to be very low (*R*^2^ = 0.096, *p* = 0.56; Fig. 4E). Having established that no correlation existed between these covariates, their correlation to length of ICU stay was examined in our patient cohort using multiple regression. Correlation within the model was found to be negligible (*R*^2^ = 0.2467, *p* = 0.31). We thus concluded that, although rare variants in the type I IFN pathway were enriched in critical COVID-19 patients, neither PRS nor a binary variable indicating the presence of rare type I IFN pathway variants, nor a combination of the two, predicted the duration of ICU treatment for COVID-19.

### Functional Interrogation of Type I IFN Pathways

**Fig. 5:**
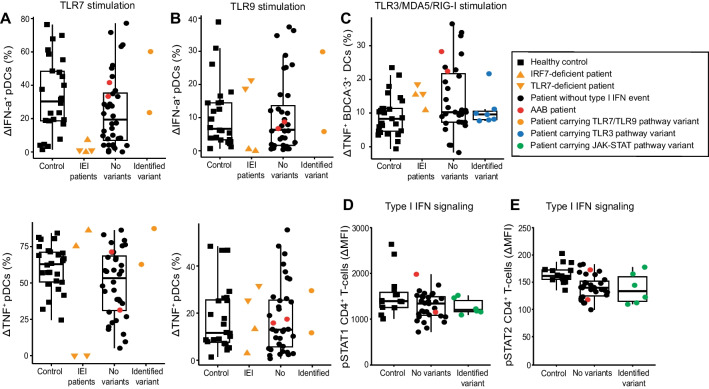
Stimulation of type I IFN pathways in healthy controls, IEI patients, and ICU COVID-19 patients. **A**, **B** pDCs responses after **A** TLR7 stimulation with imiquimod or **B** TLR9 stimulation with ODN. Cells expressing IFN-α (upper) and TNF (lower) were quantified by flow cytometry. In addition to healthy controls, four IEI patients were also included who had been diagnosed with IRF7 or TLR7 deficiency. **C** DCs response after TLR3 stimulation with poly(I:C). Cells expressing TNF were quantified. **D**, **E** CD4.^+^ T cell responses to stimulation with IFN-α. Plots show the quantification after stimulation of **D** pSTAT1 and **E** pSTAT2

As very rare variants in type I IFN responses (TLR7, TLR9, and TLR3 pathways) were identified in nine of 38 patients (23.7%) in our cohort, we developed an in vitro flow cytometry assay for the sensitive detection of IFN-α production. Cryopreserved PBMCs were stimulated with agonists to TLR7 (imiquimod) or TLR9 (CpG oligodeoxynucleotide [ODN]), known to induce strong IFN-α and TNF expression by pDCs. Patient pDC numbers in the blood were within a healthy range (Supplementary Fig. 7A). As a positive control for the detection of defective cytokine responses, we compared analysis to patients previously diagnosed with IEI in *IRF7* or *TLR7* (Fig. 5A–C). These patients demonstrated the expected pattern of pDC responses considering IRF7 is required for IFN-α but not TNF production with both TLR7 and TLR9 stimulation. In contrast, in cells from patients with TLR7 deficiency, TLR7 responses were abolished but responses to TLR9 were unaffected (Fig. 5A, B). Thus, we hypothetically increased the chance to also identify structural or non-coding variants, which are more challenging to predict from genomic data. Comparing healthy controls to patients from our cohort with identified variants in the type I IFN production pathway, and patients with no relevant variants, we did not observe any significant differences in IFN-α or TNF production between the groups. This suggests that the heterozygous expression of variants in genes associated with type I IFN production by two of our patients did not affect protein function sufficiently to cause a functional defect. Patient PBMCs were also stimulated with a poly(I:C), a TLR3/MDA5/RIG-I agonist demonstrating redundancy in stimulation of blood leukocytes [74]. As expected, poly(I:C) stimulation was not significantly different between controls and patient groups in TNF expression by BDCA-3^+^ dendritic cells (DCs) (Fig. 5C).

**Fig. 6:**
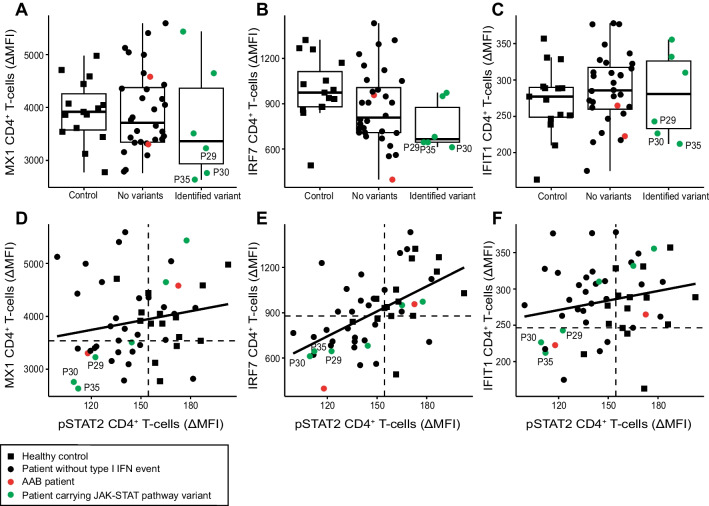
Stimulation of interferon-stimulated genes in healthy controls and ICU COVID-19 patients. **A**–**C** CD4^+^ T-cell responses after stimulation with IFN-α, measuring increase in protein expression of **A** MX1, **B** IRF7, and **C** IFIT1. **D**–**F** Correlation in CD4.^+^ T-cell responses following stimulation with IFN-α, between pSTAT2 increase and difference in protein expression of **D** MX1, **E** IRF7, and **F** IFIT1

Our genetic analysis revealed that five of the 38 patients (13.2%) harbored very rare variants in components of the signaling pathway downstream of type I IFN. Additionally, P30 carried a rare variant (p.Thr204Arg; MAF = 0.004, CADD = 24.0) in *IFNAR2* in a homozygous state. This was the only rare homozygous variant identified in a gene of interest. To assess whether these variants had a functional effect, cryopreserved PBMCs from healthy controls and patients were stimulated with IFN-α2 and assessed for upregulation of pSTAT1 and pSTAT2 (Fig. 5D–E). Upregulation of pSTAT1 and pSTAT2 had a correlation of 0.55 in CD4^+^ T cells (Supplementary Fig. 7B). Induction in CD8^+^ T cells was also examined as an internal control and correlated with the CD4^+^ T cell responses (Supplementary Fig. 6A, 6B). Examination of CD4^+^ T cells demonstrated similar levels of induction of pSTAT1 upon IFN-α stimulation in healthy controls compared to most patients with no identified relevant variants. However, pSTAT2 induction was significantly lower in patients than in healthy controls (*p* = 0.0001, Welch *T*-test), and three patients with identified *IFNAR1* or *IFNAR2* variants—P29, P30, and P35—had pSTAT2 responses below the range in healthy controls. To further interrogate type I IFN signaling in critical COVID-19 patients, we quantified protein expression of interferon-stimulated genes (ISGs) MX1, IRF7, and IFIT1 by flow cytometry (Fig. 6A–C). Although there was no significant difference between the patient and control groups, P29, P30, and P35 all had low expression of ISGs, correlating with low pSTAT2 induction (Fig. 6 D–F), suggesting that their interferon receptor gene variants may confer reduced function compared to wildtype protein.

### Genomic Screening for Variants in Other IEI Genes

**Fig. 7:**
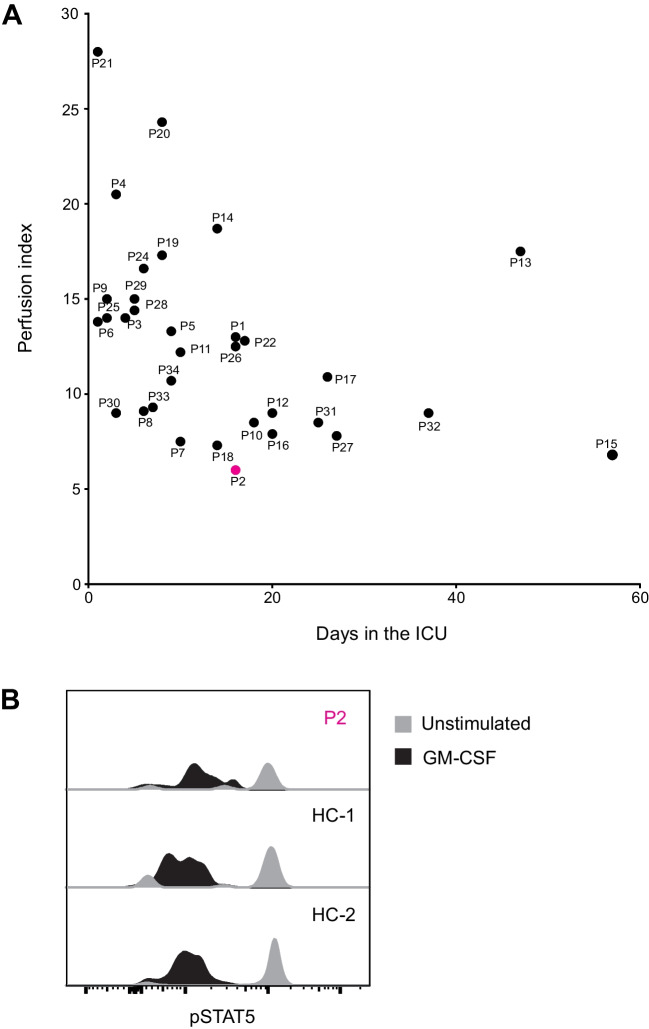
Functional investigation of GM-CSF signaling in patient P2 with low gas exchange rate in the lungs. **A** Perfusion index (arterial oxygenation (kPa)/supplied oxygen) observed in patients during their time in the ICU against the total number of days spent in ICU. Patient identity is indicated by text. Patients positive for autoantibodies are in red, and the patient with the lowest perfusion index is indicated in purple. **B** GM-CSF signaling of blood-derived monocytes from patient P2 and two healthy adult controls. Plots show intracellular staining of phosphorylated STAT5 (pSTAT5) in unstimulated and GM-CSF stimulated cells, as indicated

While efforts within the field of IEI research have identified susceptibility loci in the type I IFN pathway, GWAS studies link other pathways to life-threatening COVID-19 susceptibility. To explore other immune genes potentially implicated in life-threatening COVID-19, we identified very rare variants (MAF < 0.001) in known IEI loci using the clinical characterization of each patient to inform variant selection for further investigation. Analysis of IEI-associated genes revealed a very rare variant in *CSF2RA*, c.940G > A (p.Glu314Lys; CADD score = 13.2, GnomAD frequency = 0.00004), in P2, the patient with the longest stay in ICU and who had required prolonged mechanical ventilation with high inspiratory fraction of oxygen (Fig. 7A), ultimately culminating in extracorporeal membrane oxygenation (ECMO). Locus examination revealed a second *CSF2RA* variant, c.491G > A (p.Arg164Gln; CADD score = 12.9, GnomAD frequency = 0.0051), categorized as rare. *CSF2RA* encodes the α-subunit protein of the heterodimeric receptor for granulocyte–macrophage colony-stimulating factor (GM-CSF). CSF2RA-deficient patients have previously been reported with pulmonary insufficiency during infections, including *Mycoplasma pneumoniae* and influenza due to defective macrophage clearing of surfactant in the alveoli, interfering with gas exchange in the lungs [84]. Variant co-occurrence estimation in GnomAD indicated a 100% probability that these variants occur in different haplotypes, with no individuals identified in the database who carried both. We thus inferred that the patient likely is compound heterozygous for the two variants and sought to functionally test the GM-CSF signaling pathway in cryopreserved PBMCs from the patient. PBMCs were stimulated with GM-CSF before flow cytometric analysis for pSTAT5 induction, which is downstream of GM-CSF signaling. Gating on monocytes which express high levels of the GM-CSF receptor, we found that P2 had a similar pattern of pSTAT5 induction as healthy controls (Fig. 7B). This finding suggests that the variants in *CSF2RA* identified in P2 do not confer an impairment in GM-CSF signaling. Furthermore, we observed a novel *NLRC4* c.1003A > C (p.Met335Leu; CADD score = 18.2) variant in P14. Given the autosomal dominant inheritance mode of GoF variants in *NLRC4* and the previous description of critical COVID-19 in an *NLRC4* patient [85], this variant was especially notable and could be investigated further in the future.

In summary, we did not identify impairments in novel immune signaling pathways as potential causes of critical COVID-19. Nonetheless, our catalogue of rare variants represents a resource for further genetic investigations.

## Discussion

We performed a comprehensive clinical, genetic, and immunological functional screening of 38 young adult ICU patients with minimal known risk factors for COVID-19. While critical COVID-19 could be explained by type I IFN autoantibodies in two patients, genetic and functional screens as well as specific investigations did not identify additional patients with monogenic IEI. However, seven deceased patients who met study criteria but were not possible to include could have carried type I IFN autoantibodies or IEI-causing variants.

Autoantibodies to type I IFN are more prevalent in patients with severe COVID-19 and explained critical COVID-19 in two cases from our cohort. Our rate of 5.3% (2/38) is consistent with the rate of type I IFN autoantibodies discovered in younger, critically ill COVID-19 patients (5.0% and 6.8% of critically ill patients under 40 years or between 40 and 49 years of age, respectively) [68]. Notably, neither of our patients with type I IFN autoantibodies carried rare variants in the non-canonical NFκB signaling pathway [86]. In one of our patients, type I IFN autoantibodies likely arose due to thymoma of type AB (Masaoka grade IIa, pT2), as previously described [87, 88]. Thymomas are associated with development of a variety of antibody-mediated autoimmune disorders and paraneoplastic syndromes, reflecting dysregulation of T cell selection in the thymus [89]. Thus, autoantibody screening may identify patients that will benefit from plasmapheresis [52].

With respect to genetic causes of critical COVID-19, we observed a significant trend towards enrichment of very rare missense variants in the type I IFN signaling pathway in our cohort when adjusted for ancestry PCs and compared to a set of genes not associated with viral immunity but with similar size and constraint metrics. In this cohort, no patients displayed functional defects as severe as those identified in TLR7, IRF7, or type I IFN receptor deficiency [41, 43–45]. Although there was no significant difference between the responses of carriers and non-carriers of a very rare variant in the IFN pathways, low type I IFN signaling and ISG expression were observed in two patients that also carried very rare heterozygous variants in *IFNAR1* or *IFNAR2*, as well as one patient with a rare homozygous variant in *IFNAR2*. Importantly, the normal responses observed in other patients carrying very rare predicted damaging heterozygous variants illustrate how genomics studies lacking functional validation may lead to over-interpretation of the significance of certain variants. Leukocyte assays such as these can thereby direct biochemical testing of specific variants that can determine the exact functional consequences, potentially validating deleterious disease-causing variants.

For certain genes, e.g*.*, *TLR3* [74], impairments of specific rare variants may not be sensed in leukocyte assays but would be apparent in other cell types or upon assessment in more biochemical systems. Furthermore, another limitation of our study was the use of cryopreserved cells, necessitated by the scattered collection of samples and the effort to limit inter-experimental variability. In our experience, as documented in the literature, pDC numbers and function are reduced following cryopreservation [90, 91]. Nonetheless, we believe our results are interesting as a reference for clinical pDC defect diagnostics, and importantly, our assays on cryopreserved material did detect defects in confirmed IEI patients with TLR7 and IRF7 deficiency, albeit with lower sensitivity than assays using freshly isolated cells [39, 43].

The small size of our cohort increases random chance that the previously stated figure of 4.8% of cases are predisposed by type I IFN pathway-related IEI is accurate; none might be represented in our study. Investigating other mechanisms of susceptibility by applying PRS adjusted by ancestry PCA, we did not observe significant enrichment of risk variants in our cohort. We considered the use of a population database to be valuable as it facilitated PC calculations and the use of a larger control dataset than used in previous studies, despite lack of COVID-19 phenotype for the participants [92–94]. Nonetheless, PRS on common risk variants or combined genetic analyses of PRS and rare variants did not provide sufficient predictive power to be useful for clinical assessments of patients with critical COVID-19. Thus, the predictive value of genome analyses is relatively modest even in a selected cohort of previously healthy young adult critical COVID-19 patients.

Besides type I IFN pathway defects, other IEIs may predispose to life-threatening COVID-19. We functionally tested GM-CSF signaling in monocytes from a patient with likely biallelic missense variants in *CSF2RA* (encoding the GM-CSF receptor α-subunit) as CSF2RA-deficient patients are susceptible to severe pulmonary infections [73]. However, the monocytes were not functionally impaired, further highlighting the importance of testing putative disease-causing pathways in patients. Additionally, several HLH genes associated with defective cytotoxic responses have been investigated in the context of COVID-19 [95, 96], including a 52-year-old patient carrying a GoF *NLRC4* variant who had previously experienced recurrent HLH episodes [85]. We identified a novel *NLRC4* missense variant in one patient, potentially warranting further functional investigations of the role of NLRC4 in inflammatory responses to viral infection.

In conclusion, genetic and immunological characterization of a stringently defined, young, previously healthy critical COVID-19 patient group confirmed the utility of autoantibody screening for explaining critical patients and directing therapy. Our genomic analyses uncovered an increased incidence of very rare variants in the type I IFN pathway when compared to controls and adjusted for ethnicity. In three out of six instances, such a genetic variant correlated with low pSTAT and ISG protein upregulation, highlighting the need for tailored biochemical assays of variants to better determine causality. Sensitive leukocyte assays may aid screening of sporadic patients for further genetic investigation, but interpretation of causality from genetic findings still requires stringent biochemical assays.

### Supplementary Information

Below is the link to the electronic supplementary material.

Supplementary file1 (PDF 5113 KB)

Supplementary file2 (DOCX 357 KB)

## Data Availability

The datasets generated during the study are available from the corresponding author on reasonable request.
