# Supplementary material for: Evaluation of Genetic or Cellular Impairments in Type I IFN Immunity in a Cohort of Young Adults with Critical COVID-19
Source: J Clin Immunol. 2024 Jan 17;44(2):50. doi: 10.1007/s10875-023-01641-1 (PMC10794435; doi:10.1007/s10875-023-01641-1)
Supplement: Supplementary file 1 — Supplementary file1 (PDF 5113 KB) [file 10875_2023_1641_MOESM1_ESM.pdf]

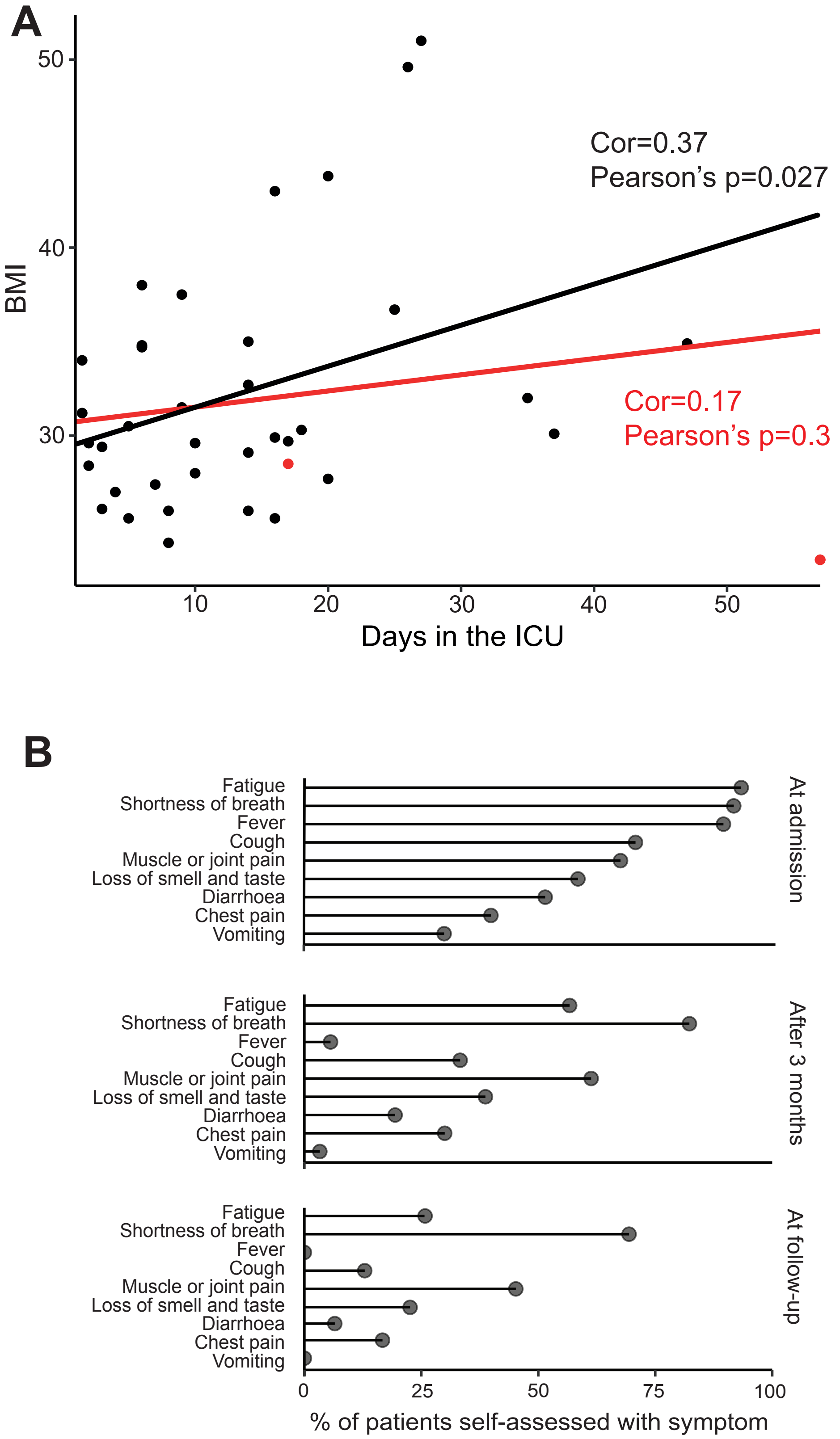

Supplementary Figure 1. Covill, Sendel, Campbell, *et al*.

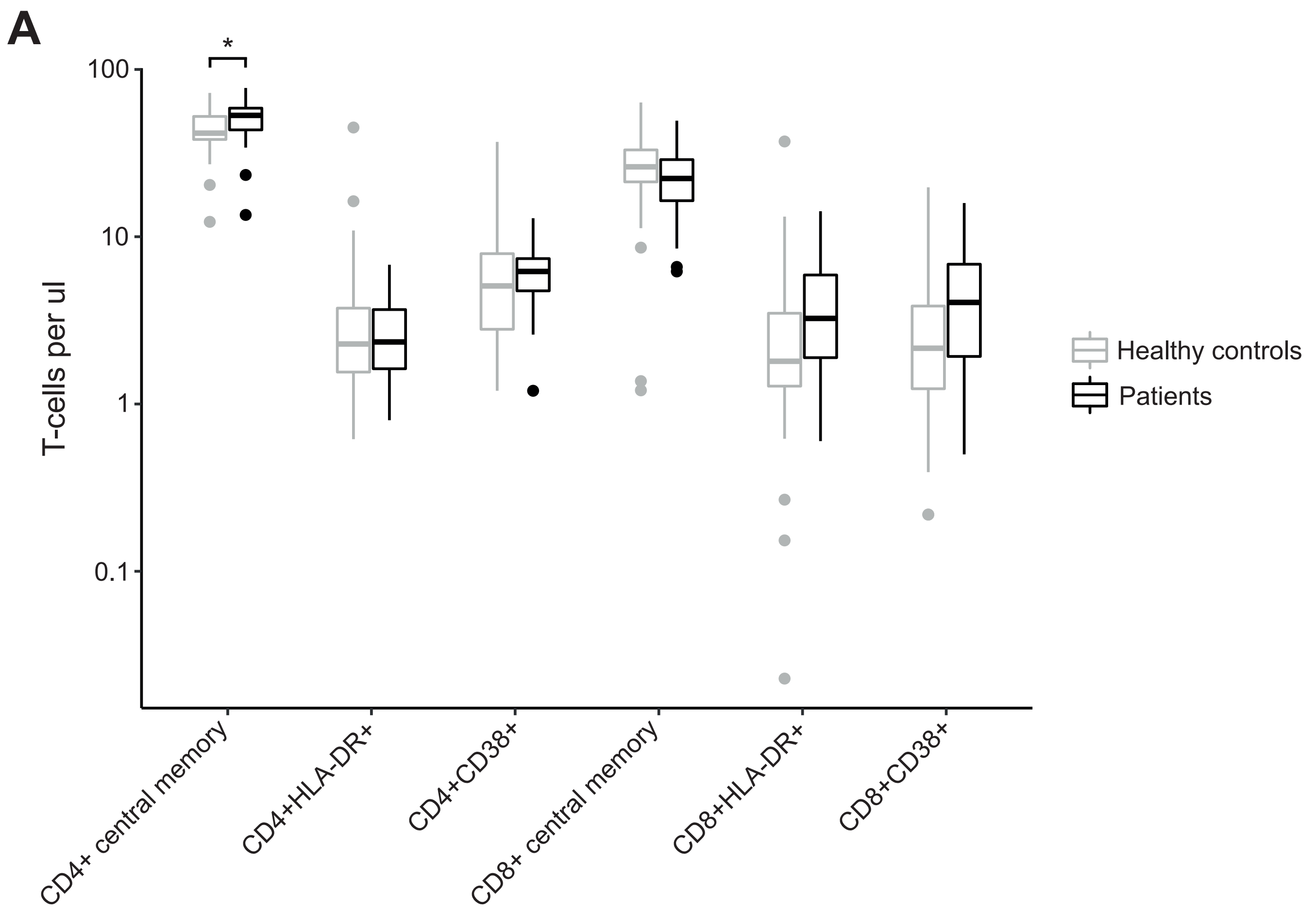

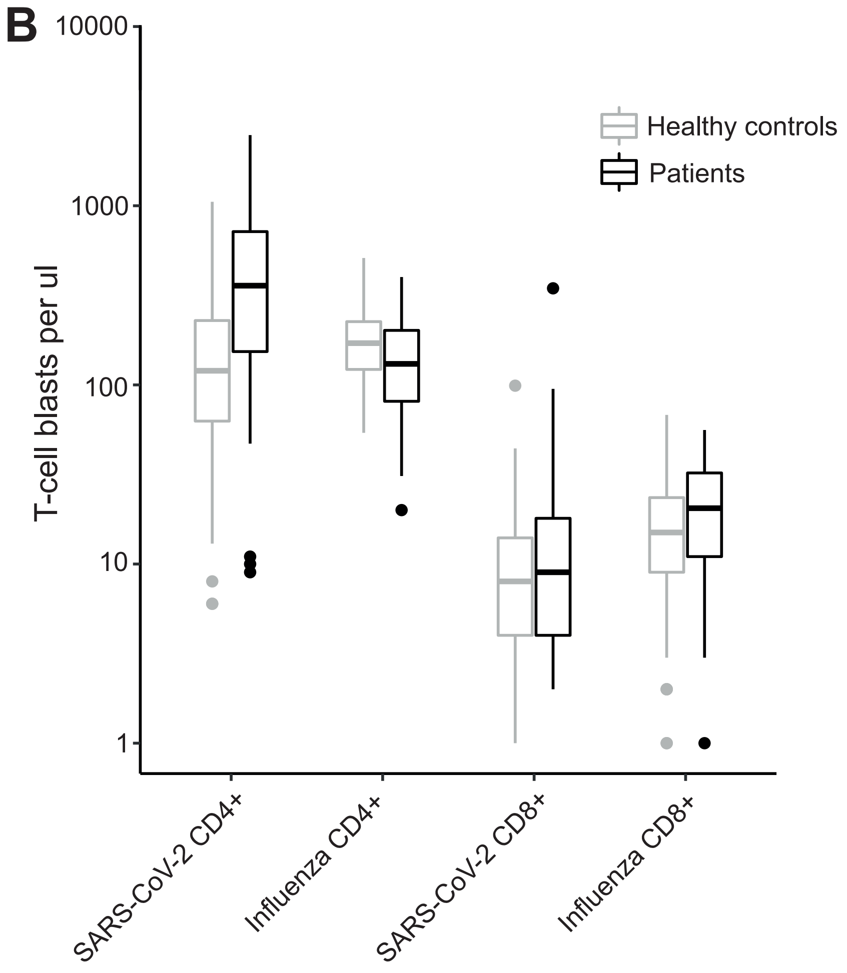

Supplementary Figure 2. Covill, Sendel, Campbell *et al*.

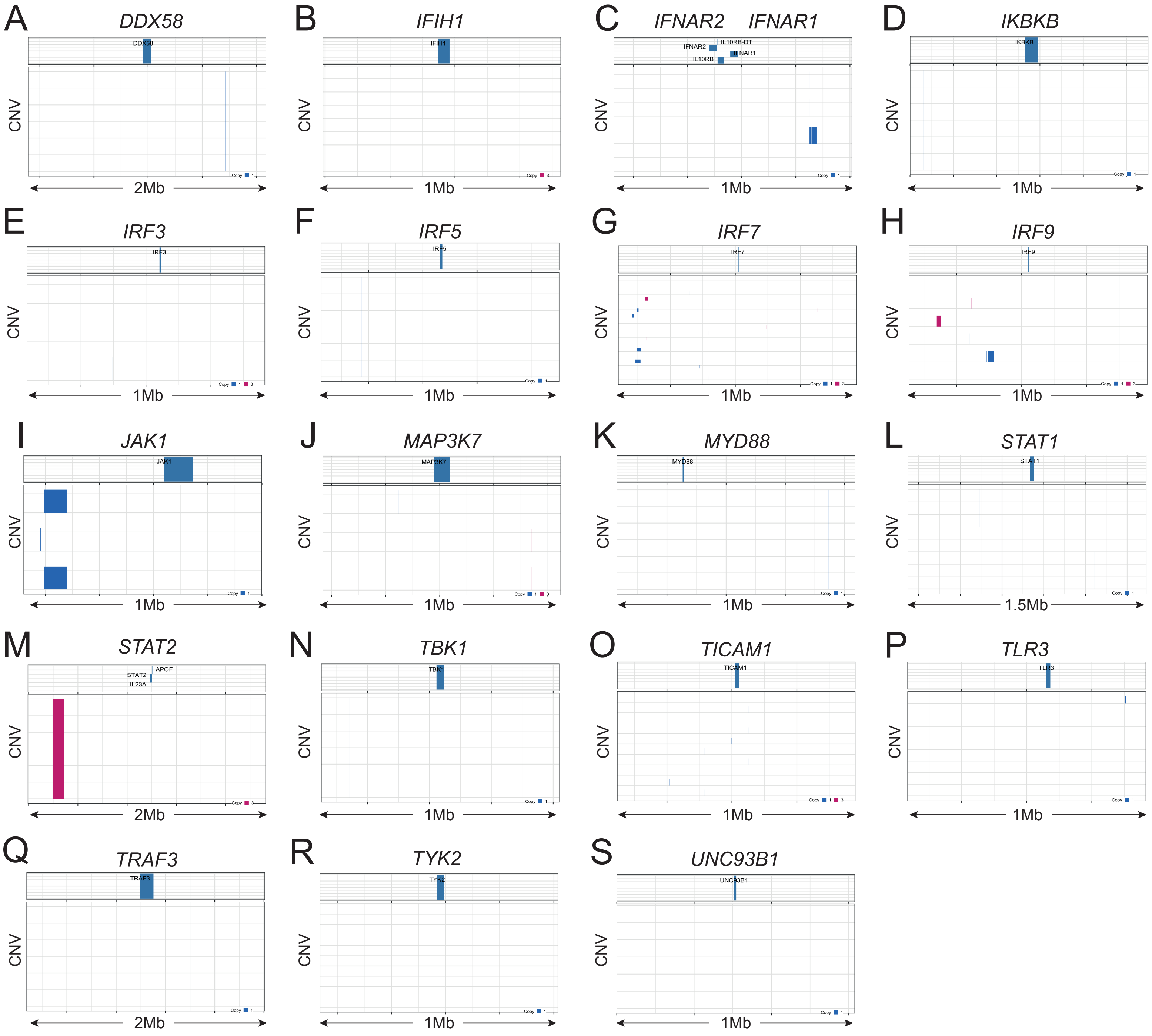

Supplementary Figure 3. Covill, Sendel, Campbell *et al.*

A

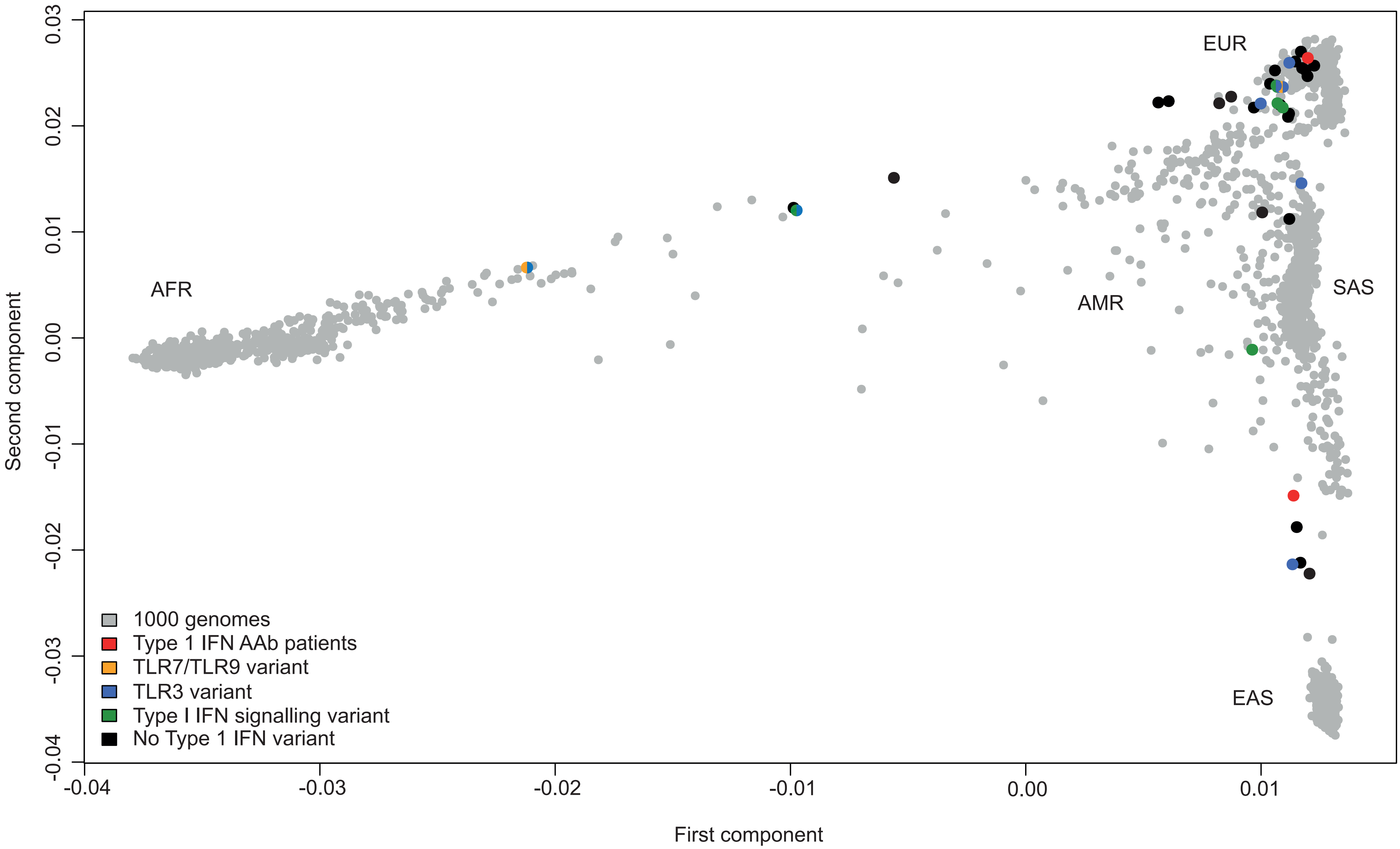

Supplementary Figure 4. Covill, Sendel, Campbell *et al*.

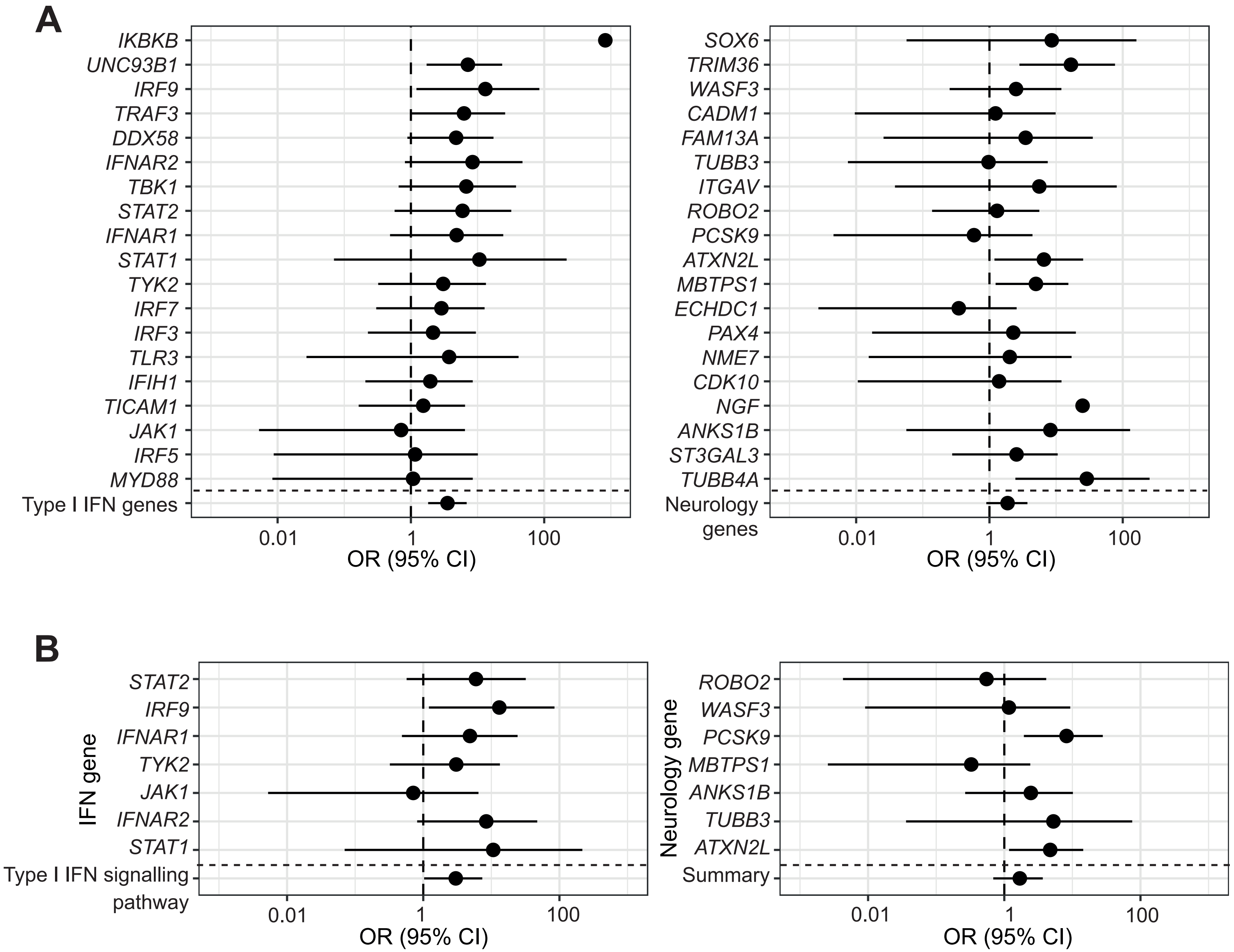

Supplementary Figure 5. Covill, Sendel, Campbell *et al*.

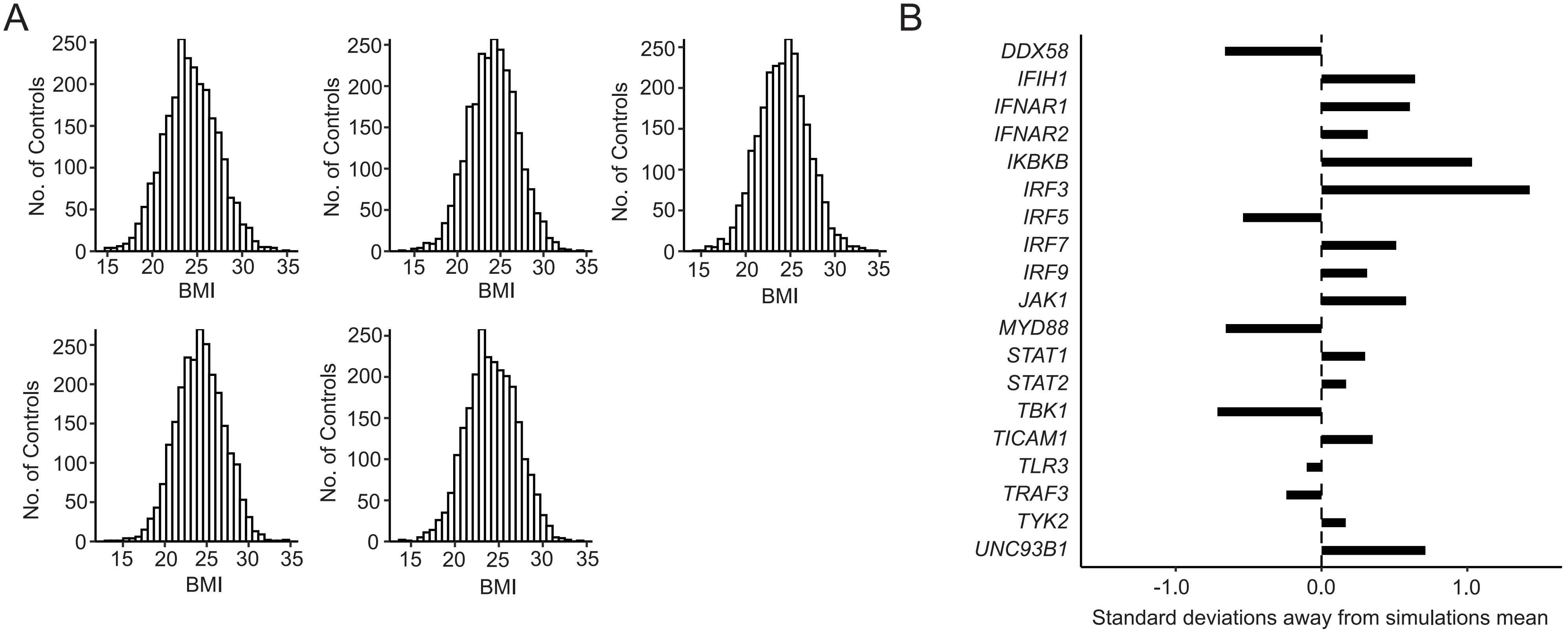

Supplementary Figure 6. Covill, Sendel, Campbell *et al*.

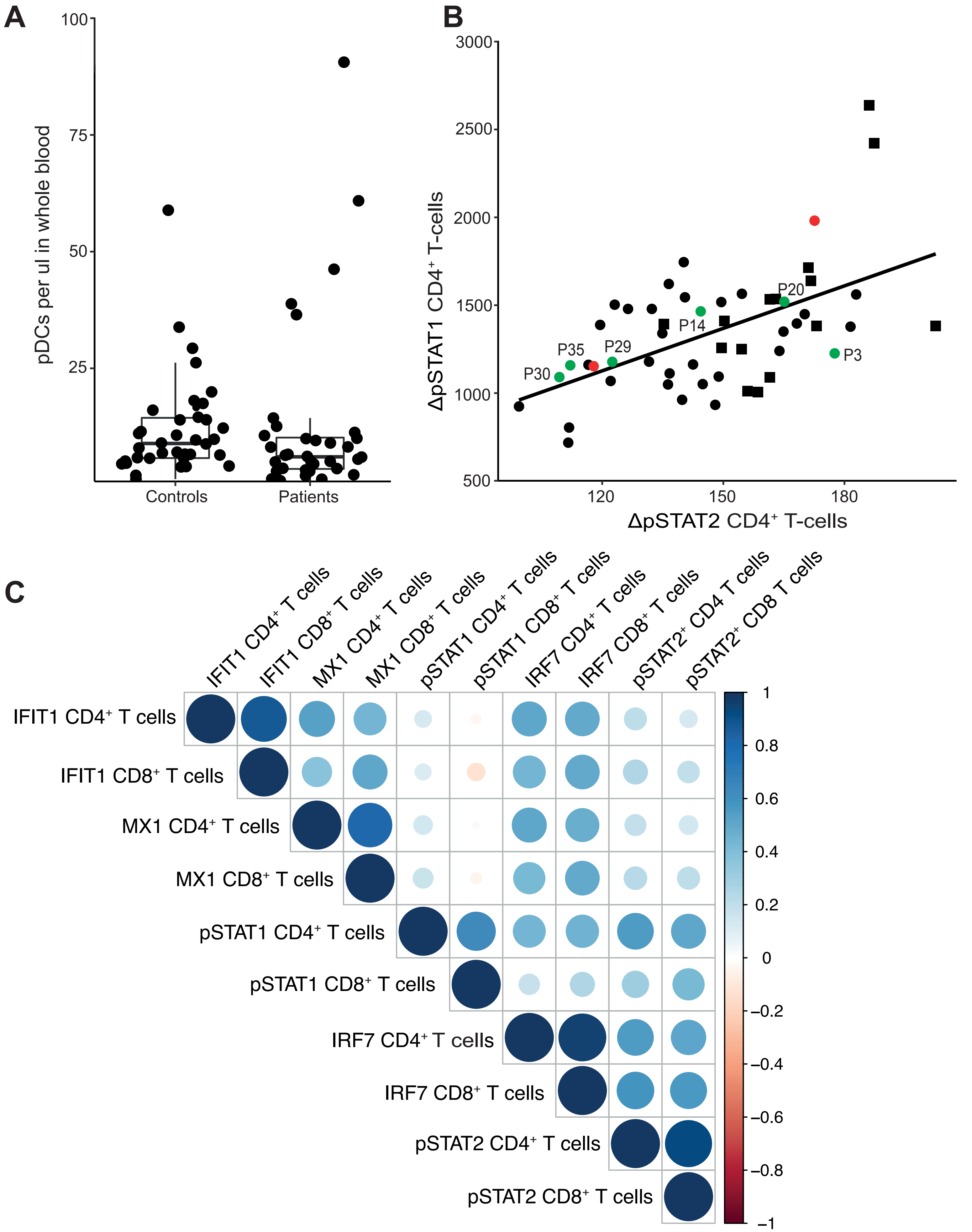

Supplementary Figure 7. Covill, Sendel, Campbell *et al*.
