# Supplementary material for: Evaluation of Genetic or Cellular Impairments in Type I IFN Immunity in a Cohort of Young Adults with Critical COVID-19
Source: J Clin Immunol. 2024 Jan 17;44(2):50. doi: 10.1007/s10875-023-01641-1 (PMC10794435; doi:10.1007/s10875-023-01641-1)
Supplement: Supplementary file 2 — Supplementary file2 (DOCX 357 KB) [file 10875_2023_1641_MOESM2_ESM.docx]

**SUPPLEMENTARY TEXT**

**Detailed material and methods**

*ICU data collection*

Clinisoft software is used for continuous collection of medical records for patient treatment in the ICU. For other hospital medical records, Take Care software is employed.

*IEI patient inclusion*

Four previously reported patients with diagnosed deficiencies in TLR7 or IRF7 were included in cellular assays and autoantibody screening as internal controls [1,2]. These patients did not meet the selection criteria to be included in the study however. Biological material from deceased patients matching the selection criteria was unavailable.

*Self-assessed symptoms*

Patients were asked to fill in forms for self-assessment of symptoms at hospital admission, 3 months after discharge, as well as at time of follow-up. Cohort proportions experiencing each symptom were calculated from positive and negative responses for each symptom.

*Blood samples*

Blood samples were collected in heparin-treated tubes from patients or healthy blood bank donors (Karolinska University Hospital). PBMCs were obtained by density gradient centrifugation (Lymphoprep; STEMCELL Technologies) and resuspended in complete medium (RPMI 1640 with GlutaMAX [Gibco] supplemented with 10% fetal bovine serum [FBS, Thermo Fisher Scientific]) for further analysis.

*Follow-up clinical visits*

Cohort patients were enrolled at the Immunodeficiency Unit outpatient clinic at Karolinska University Hospital, Stockholm, Sweden. Medical charts were examined for the hospitalization period for COVID-19 and ensuing convalescence. Patient history was examined for previous infections, immunological disease, and family history of immunodeficiency. Blood sample analyses were performed during follow-up visits including blood counts, immunoglobulin classes and subclasses (including positivity for SARS-CoV-2 spike protein antibodies), complement factors, and vitamin D levels.

*FASCIA*

Whole blood was collected in Na-heparin tubes and stimulated *in vitro* with mitogens and specific antigens, as previously described [3,4]. T- and B-lymphocyte activating and proliferative ability was assessed by flow-cytometric measure of blast formation 1 week after stimulation. Mitogens used include pokeweed mitogen, concanavalin A, and Staphylococcal enterotoxin A and B. Specific antigens used include SARS-CoV-2, Tetravac (tetanus, diphtheria, polio and pertussis vaccine), PPD, Candida albicans, Vaxigrip (influenza vaccine), Varicella zoster, cytomegalovirus, and Herpes simplex virus.

*Whole genome sequencing*

Genomic DNA was extracted from whole blood using the QIAamp DNA Mini Kit (Qiagen). Whole-genome sequencing (Illumina) was performed according to the MIP rare disease pipeline described by the Genomic Medicine Center Karolinska-Rare Diseases (GMCK-RD) [5].

*Ancestry analysis*

BCFtools was used to merge 1kGP and study genomic data. Plink2 was used to prune out variants with MAF <1% and regions of linkage disequilibrium, and perform the principle components analysis (PCA). The PCA was visualised using R (version 4.1.1).

*Polygenic risk score quality controls*

Quality controls were performed on the target dataset to remove SNPs with MAF <0.01 and duplicate SNPs. SNPs with genotyping rate less <1%, MAF <1%, or departure from the Hardy-Weinberg Equilibrium were removed. Samples were further tested for excessively high or low heterozygosity, though none were excluded. Regions of linkage disequilibrium were pruned.

*Filtration of GnomAD variants*

Genes with robust experimental evidence demonstrating association with type I IFN production and signaling were identified from a literature search [6–11]. Of these genes, we selected those which had been linked to monogenic disease in humans [12–24]. We also selected genes associated with handedness, but met the approximate size and constraint metrics of each of the type I IFN genes included in the study [25,26]. Variants with MAF <0.001 in IFN and PID genes were extracted from the GnomAD database.

*Copy number variant detection*

Structural and copy number variants were processed and analysed as described by the GMCK-RD rare disease pipeline. Overlapping variation with our genes of interest was mapped for visualization using the HandyCNV R package [27].

*Variant identification in our cohort*

Variants annotated exonic or splice variant with MAF <0.001 in IFN pathways and PID genes in the GnomAD database were extracted from vcfs using BCFtools. Variants with GQ score <50 were excluded. Variants with predicted pathogenicity (CADD score above a 99% Mutation Significance Cut-Off) or predicted loss-of-function were included in subsequent analyses [28,29].

*Gene burden testing*

R package logistf was used to carry out gene burden testing. Specifically, a penalized likelihood ratio test was performed on the set of variants present in each gene in our cohort and in the 1kGP. The first four PCs of the ancestry PCA were used as covariates to account for the ethnicities in our cohort being poorly represented in GnomAD.

*Pathway burden testing*

To test overall pathway burden, given some patients had multiple rare variants identified in a single pathway, variant counts were summed in our cohort and in the 1kGP and analyzed by penalized likelihood ratio test using logistf. The first four PCs of the ancestry PCA were used as covariates.

*Sensitivity analysis*

Body mass index (BMI) was randomly simulated for 2504 1kGP participants according to a normal distribution with center between 20-25. Mean and standard deviation were calculated for the odds ratios derived from application of this co-variate to the Firth’s penalized likelihood ratio test.

*Toll-like receptor stimulation of dendritic cells*

Thawed PBMCs from cryopreserved samples (1–1.5 × 10^6^ cells/well) were stimulated in complete medium in flat-bottom 96-well plates, as previously described [2]. Freshly thawed cells were used for ODN and poly(I:C) stimulations, while cells were first rested overnight before imiquimod stimulations, with respective unstimulated controls for both conditions. Cells were stimulated with 4 µg/ml imiquimod (Alfa Aesar), 2 μM CpG ODN 2336 (InvivoGen), 10 μg/ml poly(I:C) (Miltenyi Biotec) or left unstimulated for 6 hours in total at 37°C with 5% CO_2_. The addition of GolgiPlug (BD Biosciences) was optimised for each set of conditions. GolgiPlug was added after 1.5 hours of stimulation for imiquimod, 3 hours after stimulation for poly(I:C), or 4.5 hours after stimulation for ODN.

At the end of the assay, cells were collected and incubated with CF430 viability dye (Biotium) and cell-surface antibody cocktails in FACS buffer (Phosphate buffer saline [PBS] supplemented with 1% FBS and 10 mM EDTA) at room temperature for 20 min. Cells were then fixed by incubation in 2% formaldehyde (Thermo Fisher Scientific) at room temperature for 15 min. The cells were permeabilised by washing twice in BD Perm/Wash Buffer (BD Biosciences) and stained for intracellular markers in Perm/Wash at room temperature for 30 min. Cells were then washed in Perm/Wash and finally resuspended in FACS buffer for acquisition on a BD FACSymphony flow cytometer (BD Biosciences) and analysis with FlowJo software (v10; BD Biosciences).

*ISG detection in PBMCs*

Thawed PBMCs from cryopreserved samples (4 × 10^5^ cells/well) were rested overnight in complete medium in low-attachment round-bottom 96-well plates. The next day cells were stimulated with 1000 U/ml IFN-α-2a (R&D Systems) or kept in medium for unstimulated controls, and 24 hours later harvested for flow cytometry analysis. Cells were stained with AF700 NHS ester viability dye (Thermo Fisher) and cell-surface antibody cocktails in FACS buffer at room temperature for 20 min. Next, cells were fixed by incubation in 2% formaldehyde (Thermo Fisher Scientific) at room temperature for 15 min, and then permeabilised with 0.1% Triton X-100 (Sigma Aldrich) in FACS buffer for 5 min. Subsequently cells were stained for intracellular markers and finally resuspended in FACS buffer for acquisition on a BD FACSymphony flow cytometer (BD Biosciences) and analysis with FlowJo software (v10; BD Biosciences).

*Quantification of phosphorylated STAT1*

For autoantibody screening, previously frozen patient plasma isolated by centrifugation from heparin treated blood was thawed. Plasma was diluted to 10% in RPMI medium without FCS, and incubated with 1000 U/ml IFN-α-2a (R&D Systems) for 20 mins at 37°C. The plasma/cytokine mix was then added to 4 x 10^5^ healthy control PBMCs in a 96 well U-bottom plate and incubated for 15 mins at 37°C. Assays were performed with biological duplicate PBMCs, and healthy control plasma was used as a negative control. For assessment of type I IFN signaling in patient cells, thawed PBMCs from cryopreserved samples (4 x 10^5^ cells/well) were resuspended in complete RPMI and stimulated with 1000 U/ml recombinant human IFN-α-2a (R&D Systems), 100 ng/ml recombinant human IFNg (PeproTech), or left unstimulated for 15 mins at 37°C.

For both assays, cells were subsequently immediately fixed by transfer into an equal volume of warm 4% formaldehyde (Thermo Fisher Scientific) in PBS at 37°C for 12 mins. Cells were then washed in cold FACS buffer before permeabilisation with 0.1% Triton X-100 (Sigma Aldrich) in PBS on ice for 10 min. After washing twice in PBS, samples were barcoded by transferring into a 96 well V-bottom plate containing complexed 4x4 serial dilutions of A350 dye and Pacific Blue dye (both Thermo Fisher Scientific) and incubated at room temperature for 15 mins. Subsequently barcoded cells were washed twice in FACS buffer and pooled. The samples were then permeabilised with 2 ml cold methanol (Milipore) and kept at -20°C overnight. The next day samples were washed twice in FACS buffer before staining with a Phosflow antibody cocktail in FACS buffer at room temperature for 45 mins. Samples were given two final washes and resuspended in FACS buffer for acquisition on a BD FACSymphony flow cytometer (BD Biosciences) and analysis with FlowJo software (v10; BD Biosciences).

*Antibodies for flow cytometry*

The AF647–anti-human MX1 (clone EPR19967) antibody was obtained from Abcam. The following antibodies from BD Biosciences were used: BUV805–anti-human CD3 (clone UCHT1), BV711–anti-human CD11c (clone B-ly6), APC-Cy7–anti-human CD19 (clone SJ25C1), BUV395–anti-human CD56 (clone B159), PE-Cy7–anti-human CD95 (clone DX2), AF647–anti-human pSTAT1 (pY701) (clone 4a), AF647–anti-human pSTAT5 (pY694) (clone 47/Stat5(pY694)), FITC–anti-human SYK (clone 4D10), and PE–anti-human TNF (clone MAb11). The following antibodies were obtained from BioLegend: BV421–anti-human CCR7 (clone G043H7), BV785–anti-human CD3 (clone OKT3), BV650–anti-human CD4 (clone RM4-5), BV711–anti-human CD8a (clone SK1), BV510–anti-human CD14 (clone M5E2), BV510–anti-human CD19 (clone HIB19), PE-Cy7–anti-human CD45RA (clone HI100), PE-Cy7–anti-human CD123 (clone 6H6), BV421–anti-human CD303 (BDCA-2; clone 201A), BV785–anti-human HLA-DR (clone L243), FITC–anti-human IFN-γ (clone 4S.B3), and PE–anti-human IRF7 (clone 12G9A36). The AF488–anti-human IFIT1 (clone D2X9Z) antibody was procured from Cell Signaling Technology. The Qdot605 or Qdot655–anti-human CD4 (clone S3.5) and Qdot605–anti-human CD8 (clone 3B5) antibodies was obtained from Thermo Fisher Scientific. The following antibodies from Miltenyi Biotec were used: FITC–anti-human CD141 (BDCA-3; clone AD5-14H12) and APC–anti-human IFN-α (clone LT27:295).

*Variant co-occurrence analysis*

Variant co-occurrence for two variants in *CSF2RA* was carried out using the inferred phasing data available in the GnomAD v2 browser (https://gnomad.broadinstitute.org/news/2021-07-variant-co-occurrence-phasing-information-in-gnomad/).

*GM-CSF stimulation*

Patient or healthy control PBMCs (4 x 10^5^ cells/well) were thawed and rested overnight in complete RPMI before stimulation with 20 ng/ml recombinant human GM-CSF (PeproTech) or left unstimulated for 15 mins at 37°C. Fixing, barcoding and staining was then performed as stated.

**References**

1. Asano T, Boisson B, Onodi F, Matuozzo D, Moncada-Velez M, Maglorius Renkilaraj MRL, et al. X-linked recessive TLR7 deficiency in ~1% of men under 60 years old with life-threatening COVID-19. Sci. Immunol. 2021;6(62):eabl4348.
2. Campbell TM, Liu Z, Zhang Q, Moncada-Velez M, Covill LE, Zhang P, et al. Respiratory viral infections in otherwise healthy humans with inherited IRF7 deficiency. J. Exp. Med. 2022;219(7):e20220202.
3. Lind Enoksson S, Bergman P, Klingström J, Boström F, da Silva Rodrigues R, Winerdal ME, et al. A flow cytometry-based proliferation assay for clinical evaluation of T-cell memory against SARS-CoV-2. J. Immunol. Methods. 2021;499:113159.
4. Marits P, Wikström AC, Popadic D, Winqvist O, Thunberg S. Evaluation of T and B lymphocyte function in clinical practice using a flow cytometry based proliferation assay. Clin. Immunol. 2014;153:332–42.
5. Stranneheim H, Lagerstedt-Robinson K, Magnusson M, Kvarnung M, Nilsson D, Lesko N, et al. Integration of whole genome sequencing into a healthcare setting: high diagnostic rates across multiple clinical entities in 3219 rare disease patients. Genome Med. 2021;13(1):40.
6. Platanias LC. Mechanisms of type-I- and type-II-interferon-mediated signalling. Nat Rev Immunol. 2005. p. 375–86.
7. Perry AK, Chen G, Zheng D, Tang H, Cheng G, Cheng G. The host type I interferon response to viral and bacterial infections. Cell Res [Internet]. 2005;15:407–22.
8. Pan H, Yan BS, Rojas M, Shebzukhov Y V., Zhou H, Kobzik L, et al. Ipr1 gene mediates innate immunity to tuberculosis. Nature. 2005;434:767–72.
9. Kawai T, Takahashi K, Sato S, Coban C, Kumar H, Kato H, et al. IPS-1, an adaptor triggering RIG-I- and Mda5-mediated type I interferon induction. Nat Immunol. 2005;6:981–8.
10. Sharma S, TenOever BR, Grandvaux N, Zhou GP, Lin R, Hiscott J. Triggering the interferon antiviral response through an IKK-related pathway. Science (1979). 2003;300:1148–51.
11. Zhang S-Y, Jouanguy E, Ugolini S, Smahi A, Elain G, Romero P, et al. TLR3 Deficiency in Patients with Herpes Simplex Encephalitis. Science (1979). 2007;317:1522–7.
12. Herman M, Ciancanelli M, Ou YH, Lorenzo L, Klaudel-Dreszler M, Pauwels E, et al. Heterozygous TBK1 mutations impair TLR3 immunity and underlie herpes simplex encephalitis of childhood. Journal of Experimental Medicine. 2012;209:1567–82.
13. Hernandez N, Melki I, Jing H, Habib T, Huang SSY, Danielson J, et al. Life-threatening influenza pneumonitis in a child with inherited IRF9 deficiency. Journal of Experimental Medicine. 2018;215:2567–85.
14. Del Bel KL, Ragotte RJ, Saferali A, Lee S, Vercauteren SM, Mostafavi SA, et al. JAK1 gain-of-function causes an autosomal dominant immune dys- regulatory and hypereosinophilic syndrome. Journal of Allergy and Clinical Immunology. 2017;139:2015-2016.e4.
15. Hernandez N, Bucciol G, Moens L, Le Pen J, Shahrooei M, Goudouris E, et al. Inherited IFNAR1 deficiency in otherwise healthy patients with adverse reaction to measles and yellow fever live vaccines. Journal of Experimental Medicine. 2019;216:2057–70.
16. de Jesus AA, Hou Y, Brooks S, Malle L, Biancotto A, Huang Y, et al. Distinct interferon signatures and cytokine patterns define additional systemic autoinflammatory diseases. Journal of Clinical Investigation. 2020;130:1669–82.
17. Bastard P, Manry J, Chen J, Rosain J, Seeleuthner Y, AbuZaitun O, et al. Herpes simplex encephalitis in a patient with a distinctive form of inherited IFNAR1 deficiency. Journal of Clinical Investigation. 2021;131.
18. Hambleton S, Goodbourn S, Young DF, Dickinson P, Mohamad SMB, Valappil M, et al. STAT2 deficiency and susceptibility to viral illness in humans. Proc Natl Acad Sci U S A. 2013;110:3053–8.
19. Jang MA, Kim EK, Now H, Nguyen NTH, Kim WJ, Yoo JY, et al. Mutations in DDX58, which encodes RIG-I, Cause atypical singleton-merten syndrome. Am J Hum Genet. 2015;96:266–74.
20. Pérez de Diego R, Sancho-Shimizu V, Lorenzo L, Puel A, Plancoulaine S, Picard C, et al. Human TRAF3 Adaptor Molecule Deficiency Leads to Impaired Toll-like Receptor 3 Response and Susceptibility to Herpes Simplex Encephalitis. Immunity. 2010;33:400–11.
21. Andersen LL, Mørk N, Reinert LS, Kofod-Olsen E, Narita R, Jørgensen SE, et al. Functional IRF3 deficiency in a patient with herpes simplex encephalitis. Journal of Experimental Medicine. 2015;212:1371–9.
22. Frodsham AJ, Zhang L, Dumpis U, Azizah Mohd Taib N, Best S, Durham A, et al. Class II cytokine receptor gene cluster is a major locus for hepatitis B persistence. Proc Natl Acad Sci USA [Internet]. 2006;103:9148–53. Available from: [www.pnas.orgcgidoi10.1073pnas.0602800103](http://www.pnas.orgcgidoi10.1073pnas.0602800103)
23. Cardinez C, Miraghazadeh B, Tanita K, Da Silva E, Hoshino A, Okada S, et al. Gain-of-function IKBKB mutation causes human combined immune deficiency. Journal of Experimental Medicine. 2018;215:2715–24.
24. Casrouge A, Zhang S-Y, Eidenschenk C, Jouanguy E, Puel A, Yang K, et al. Herpes Simplex Virus Encephalitis in Human UNC-93B Deficiency. Science (1979). 2006;314:308–12.
25. Cuellar-Partida G, Tung JY, Eriksson N, Albrecht E, Aliev F, Andreassen OA, et al. Genome-wide association study identifies 48 common genetic variants associated with handedness. Nat Hum Behav. 2021;5:59–70.
26. Sha Z, Pepe A, Schijven D, Carri on-Castillo A, Roe JM, Westerhausen R, et al. Handedness and its genetic influences are associated with structural asymmetries of the cerebral cortex in 31,864 individuals. Proc Natl Acad Sci USA [Internet]. 2021;118.
27. Zhou J, Liu L, Lopdell TJ, Garrick DJ, Shi Y. HandyCNV: Standardized Summary, Annotation, Comparison, and Visualization of Copy Number Variant, Copy Number Variation Region, and Runs of Homozygosity. Front Genet. 2021;12.
28. Itan Y, Shang L, Boisson B, Ciancanelli MJ, Markle JG, Martinez-Barricarte R, et al. The mutation significance cutoff: gene-level thresholds for variant predictions. Nat Methods. Nature Publishing Group; 2016. p. 109–10.
29. Kircher M, Witten DM, Jain P, O’roak BJ, Cooper GM, Shendure J. A general framework for estimating the relative pathogenicity of human genetic variants. Nat Genet. 2014;46:310–5.

**SUPPLEMENTARY DATA**

**Supplementary Table 1. Self-assessed symptom scoring for 9 common symptoms of COVID-19 taken by questionnaire at hospital admission; at 3 month check-up; and at most recent check-up (n=31).**

|  | **Shortness of breath** | | | **Cough** | | | **Chest pain** | | | **Loss of smell and taste** | | | **Diarrhoea** | | | **Vomiting** | | | **Muscle and joint pain** | | | **Fatigue** | | | **Fever** | | |
| --- | --- | --- | --- | --- | --- | --- | --- | --- | --- | --- | --- | --- | --- | --- | --- | --- | --- | --- | --- | --- | --- | --- | --- | --- | --- | --- | --- |
|  | **At admission** | **After 3 months** | **After 6 months** | **At admission** | **After 3 months** | **After 6 months** | **At admission** | **After 3 months** | **After 6 months** | **At admission** | **After 3 months** | **After 6 months** | **At admission** | **After 3 months** | **After 6 months** | **At admission** | **After 3 months** | **After 6 months** | **At admission** | **After 3 months** | **After 6 months** | **At admission** | **After 3 months** | **After 6 months** | **At admission** | **After 3 months** | **After 6 months** |
| P1 | 1 | 1 | 1 | 1 | 0 | 0 | 0 | 0 | 0 | 1 | 1 | 0 | 0 | 0 | 0 | 0 | 0 | 0 | 1 | 1 | 1 | 1 | 1 | 1 | 1 | 0 | 0 |
| P2 | 1 | 0 | 0 | 0 | 0 | 0 | 0 | 1 | 1 | 1 | 0 | 0 | 0 | 0 | 0 | 0 | 0 | 0 | 0 | 0 | 0 | 1 | 1 | 1 | 1 | 0 | 0 |
| P3 | 1 | 1 | 0 | 1 | 1 | 0 | 0 | 0 | 0 | 0 | 0 | 0 | 0 | 0 | 0 | 0 | 0 | 0 | 1 | 0 | 0 | 1 | 0 | 0 | 1 | 0 | 0 |
| P4 | 0 | 1 | 0 | 1 | 1 | 0 | 0 | 0 | 0 | 1 | 0 | 0 | 1 | 0 | 0 | 1 | 0 | 0 | 1 | 1 | 1 | 1 | 1 | 1 | 1 | 0 | 0 |
| P5 | 1 | 1 | 1 | 1 | 1 | 0 | 1 | 0 | 0 | 0 | 0 | 0 | 0 | 0 | 0 | 0 | 0 | 0 | 1 | 1 | 1 | 1 | 1 | 1 |  | 0 | 0 |
| P6 | 1 | 0 | 0 | 0 | 0 | 0 | 1 | 0 | 0 | 1 | 1 | 1 | 1 | 0 | 0 | 0 | 0 | 0 | 1 | 0 | 0 | 1 | 0 | 0 | 1 | 0 | 0 |
| P7 | 1 | 1 | 1 | 1 | 0 | 0 | 1 | 0 | 0 | 1 | 1 | 1 | 1 | 0 | 0 | 1 | 0 | 0 | 1 | 1 | 1 | 1 | 1 | 1 | 1 | 0 | 0 |
| P8 | 1 | 1 | 1 | 1 | 1 | 1 | 0 | 1 | 1 | 1 | 0 | 0 | 0 | 0 | 0 | 0 | 0 | 0 | 0 | 1 | 1 | 1 | 1 | 1 | 1 | 0 | 0 |
| P9 | 1 | 0 | 0 | 1 | 0 | 0 | 0 | 0 | 0 | 1 | 0 | 0 | 1 | 0 | 0 | 0 | 0 | 0 | 1 | 0 | 0 | 1 | 1 | 1 | 1 | 0 | 0 |
| P10 | 1 | 0 | 0 | 0 | 0 | 0 | 0 | 0 | 0 | 0 | 0 | 0 | 1 | 0 | 0 | 0 | 0 | 0 | 1 | 1 | 1 | 0 | 0 | 0 | 0 | 0 | 0 |
| P11 | 1 | 1 | 0 | 1 | 1 | 0 | 0 | 0 | 0 | 1 | 1 | 0 | 1 | 0 | 0 | 0 | 0 | 0 | 1 | 1 | 0 | 1 | 1 | 1 | 1 | 0 | 0 |
| P12 | 1 |  | 1 | 1 |  | 0 |  |  | 1 | 1 | 1 | 1 | 1 | 0 | 0 |  |  |  | 0 | 1 | 0 | 1 | 1 | 1 | 1 | 0 | 0 |
| P13 | 1 | 1 | 0 | 1 | 0 | 1 | 0 | 1 | 1 | 1 | 1 | 0 | 1 | 1 | 1 | 0 | 0 | 0 | 0 | 1 | 1 | 1 | 1 | 1 | 1 | 0 | 0 |
| P14 | 1 | 1 | 0 | 1 | 0 | 0 | 1 | 0 | 0 | 1 | 0 | 0 | 1 | 1 | 0 | 1 | 0 | 0 | 1 | 1 | 1 | 1 | 1 | 1 | 1 | 0 | 0 |
| P15 | 0 | 0 | 0 | 0 | 0 | 0 | 0 | 0 | 0 | 0 | 0 | 0 | 0 | 0 | 0 | 0 | 0 | 0 | 1 | 0 | 1 | 1 | 1 | 1 | 0 | 0 | 0 |
| P16 | 1 | 1 | 0 | 0 | 1 | 0 | 0 | 0 | 0 | 1 | 1 | 1 | 0 | 0 | 0 | 0 | 0 | 0 | 0 | 0 | 0 | 1 | 1 | 0 | 1 | 0 | 0 |
| P17 | 1 | 0 | 0 | 1 | 0 | 0 | 1 | 1 |  | 1 | 1 | 1 | 1 | 0 | 0 | 1 | 0 | 0 | 1 | 1 | 1 | 1 | 1 | 1 | 1 | 0 | 0 |
| P18 | 1 | 1 | 0 | 0 | 0 | 0 | 1 | 1 | 0 | 1 | 1 | 1 | 1 | 1 | 1 | 0 | 0 | 0 | 1 | 1 | 0 | 1 | 1 | 0 | 1 | 1 | 0 |
| P19 | 1 | 0 | 0 | 0 | 0 | 0 | 0 | 0 | 0 | 0 | 0 | 0 | 0 | 0 | 0 | 0 | 0 | 0 | 0 | 0 | 0 | 0,5 | 0,5 | 0,5 | 1 | 0 | 0 |
| P20 | 1 | 0 | 0 | 1 | 0 | 0 | 0 | 0 | 0 | 1 | 1 | 1 | 1 | 0 | 0 | 0 | 0 | 0 | 0 | 0 | 0 | 1 | 1 | 0 | 1 | 0 | 0 |
| P21 | 1 | 0 | 0 | 1 | 0 | 0 | 0 | 0 | 0 | 0 | 0 | 0 | 0 | 0 | 0 | 0 | 0 | 0 | 0 | 0 | 0 | 1 | 0 | 0 | 0 | 0 | 0 |
| P23 | 1 | 1 | 1 | 1 | 1 | 1 | 0 | 0 | 0 | 0 | 1 | 0 | 0 | 0 | 0 | 0 | 0 | 0 | 1 | 1 | 1 | 1 | 1 | 1 | 1 | 0 | 0 |
| P24 | 1 | 1 | 1 | 1 | 1 | 1 | 1 | 1 | 0 |  | 0 | 0 | 0 | 1 | 0 | 0 | 0 | 0 | 1 | 1 | 0 | 1 | 1 | 1 | 1 | 0 | 0 |
| P25 | 1 | 1 | 1 | 0 | 0 | 0 | 1 | 1 | 1 | 1 | 0 | 0 | 1 | 0 | 0 | 1 | 0 | 0 | 1 | 1 | 1 | 1 | 1 | 1 | 1 | 0 | 0 |
| P26 | 1 | 0 | 0 | 1 | 0 | 0 | 0 | 0 | 0 | 0 | 0 | 0 | 0 | 0 | 0 | 0 | 0 | 0 | 0 | 0 | 0 | 0 | 0 | 0 |  | 0 | 0 |
| P27 | 1 | 1 | 0 | 1 | 1 | 0 | 1 | 1 | 0 | 0 | 0 | 0 | 1 | 1 | 0 | 1 | 1 | 0 | 1 | 1 | 1 | 1 | 1 | 1 | 1 | 1 | 0 |
| P30 | 1 | 1 | 0 | 0 | 0 | 0 | 0 | 0 | 0 | 0 | 0 | 0 | 1 | 1 | 0 | 1 | 0 | 0 | 1 | 1 | 0 | 1 | 1 | 1 | 1 | 0 | 0 |
| P31 | 1 | 0 | 0 | 1 | 0 | 0 | 1 | 0 | 0 | 0 | 0 | 0 | 0 | 0 | 0 | 0 | 0 | 0 | 1 | 1 | 1 | 1 | 1 | 1 | 1 | 0 | 0 |
| P33 | 1 | 0 | 0 | 1 | 0 | 0 | 0 | 0 | 0 | 1 | 0 | 0 | 0 | 0 | 0 | 0 | 0 | 0 | 0 | 0 | 0 | 1 | 1 | 0 | 1 | 0 | 0 |
| P34 | 1 | 1 | 0 | 1 | 1 | 0 | 1 | 1 | 0 |  | 1 | 0 | 1 | 0 | 0 | 1 | 0 | 0 | 1 | 1 | 0 | 1 | 1 | 1 | 1 | 0 | 0 |
| P37 | 1 | 0 | 0 | 1 | 0 | 0 | 1 | 0 | 0 | 0 | 0 | 0 | 0 | 0 | 0 | 1 | 0 | 0 | 1 | 0 | 0 | 1 | 1 | 1 | 1 | 0 | 0 |
| Total | 29 | 17 | 8 | 22 | 10 | 4 | 12 | 9 | 5 | 17 | 12 | 7 | 16 | 6 | 2 | 9 | 1 | 0 | 21 | 19 | 14 | 28,5 | 25,5 | 21,5 | 26 | 2 | 0 |

**Supplementary Table 2. SNV and constraint metrics for autosomal type I IFN genes and matched selected handedness-associated genes.**

| **IFN gene** | **MSC 99%** | **Expected SNVs** | **Observed SNVs** | **pLI** | **o/e** | **Neurology gene** | **MSC 99%** | **Expected SNVs** | **Observed SNVs** | **pLI** | **o/e** |
| --- | --- | --- | --- | --- | --- | --- | --- | --- | --- | --- | --- |
| *JAK1* | 24.42 | 61,4 | 7 | 1 | 0.11 | *ANKS1B* | 2.4 | 61,1 | 7 | 1 | 0.11 |
| *STAT1* | 11.79 | 48,9 | 4 | 1 | 0.08 | *ATXN2L* | 4.02 | 54,4 | 3 | 1 | 0.06 |
| *TRAF3* | 2.45 | 28,1 | 2 | 1 | 0.07 | *CADM1* | 19.97 | 21 | 4 | 0,66 | 0.19 |
| *IFIH1* | 17.42 | 47,3 | 59 | 0 | 1.25 | *CDK10* | 29.38 | 23,2 | 24 | 0 | 1.03 |
| *IRF7* | 2.4 | 19,2 | 21 | 0 | 1.09 | *ECHDC1* | 2.39 | 13,5 | 13 | 0 | 0.96 |
| *DDX58* | 2.4 | 48,5 | 40 | 0 | 0.83 | *FAM13A* | 3.89 | 60,1 | 38 | 0 | 0.63 |
| *TBK1* | 10.63 | 43,3 | 11 | 0,08 | 0.25 | *ITGAV* | 2.92 | 62,9 | 19 | 0 | 0.3 |
| *TYK2* | 24.48 | 57,7 | 23 | 0 | 0.4 | *MBTPS1* | 4.68 | 57,4 | 24 | 0 | 0.42 |
| *TICAM1* | 0 | 0 | 0 | NA | NA | *NGF* | 25.24 | 5,7 | 0 | 0,82 | 0 |
| *TLR3* | 22.45 | 30,3 | 16 | 0 | 0.53 | *NME7* | 8.53 | 21,4 | 12 | 0 | 0.56 |
| *IRF3* | 2.67 | 17,1 | 9 | 0 | 0.53 | *PAX4* | 20.54 | 16,1 | 8 | 0 | 0.5 |
| *IFNAR1* | 2.39 | 24 | 20 | 0 | 0.83 | *PCSK9* | 6.42 | 26,9 | 26 | 0 | 0.97 |
| *STAT2* | 20.75 | 57 | 11 | 0,93 | 0.19 | *ROBO2* | 19.31 | 74,5 | 6 | 1 | 0.08 |
| *IKBKB* | 26.34 | 54,1 | 9 | 0,99 | 0.17 | *SOX6* | 4.46 | 42,6 | 4 | 1 | 0.09 |
| *IRF5* | 6.88 | 24,3 | 8 | 0,01 | 0.33 | *ST3GAL3* | 23.46 | 25,3 | 9 | 0 | 0.36 |
| *UNC93B1* | -0.0038 | 23,2 | 6 | 0,14 | 0.26 | *TRIM36* | 0.63 | 31,4 | 13 | 0 | 0.41 |
| *IFNAR2* | 6.23 | 15,1 | 10 | 0 | 0.66 | *TUBB3* | 19.63 | 24 | 18 | 0 | 0.75 |
| *MYD88* | 4.2 | 14 | 4 | 0,12 | 0.29 | *TUBB4A* | 23.96 | 13,6 | 4 | 0,11 | 0.29 |
| *IRF9* | 4.65 | 20,4 | 4 | 0,61 | 0.2 | *WASF3* | 1.5 | 19,6 | 4 | 0,54 | 0.2 |

**Supplementary Table 3. Carriership and odds ratios of the sum of very rare variants in handedness-associated genes matched to autosomal Type I IFN genes, in our patient cohort compared to individuals in the 1kGP, adjusted by first five PCs. Bold text denotes genes with p-value <0.05**

|  | **Carriers in cases** | | | **Carriers in controls** | | |  |  |  |  |
| --- | --- | --- | --- | --- | --- | --- | --- | --- | --- | --- |
| **Gene** | **Heterozygous** | **Homozygous** | **Possible compound heterozygous** | **Heterozygous** | **Homozygous** | **Possible compound heterozygous** | **Odds ratio** | **Lower CI** | **Higher CI** | **p-value** |
| *ANKS1B* | 1 | 0 | 0 | 57 | 0 | 2 | 2.548009 | 0.2761785 | 10.57104 | 0.3385715 |
| ***ATXN2L*** | **3** | **0** | **0** | **56** | **0** | **2** | **4.980636** | **1.237045** | **15.27551** | **0.026839630** |
| *CADM1* | 0 | 0 | 0 | 13 | 0 | 0 | 3.487866 | 0.02576931 | 35.51187 | 0.4798399 |
| *CDK10* | 0 | 0 | 0 | 7 | 0 | 0 | 24.96987 | 0.1493413 | 1140.527 | 0.157603657 |
| *ECHDC1* | 0 | 0 | 0 | 25 | 0 | 0 | 2.27895 | 0.01734941 | 19.79105 | 0.6178840 |
| *FAM13A* | 0 | 0 | 0 | 76 | 0 | 0 | 0.9684728 | 0.007525959 | 7.494115 | 0.9822653237 |
| *ITGAV* | 1 | 0 | 0 | 77 | 0 | 2 | 1.300142 | 0.1377843 | 5.602786 | 0.773998079 |
| *MBTPS1* | 0 | 0 | 0 | 75 | 0 | 8 | 0.3467268 | 0.00271634 | 2.557461 | 0.3793454 |
| *NGF* | 0 | 0 | 0 | 4 | 0 | 0 | 8.213819 | 0.05665885 | 128.9174 | 0.2900197 |
| *NME7* | 0 | 0 | 0 | 26 | 0 | 1 | 1.395187 | 0.0105975 | 12.05342 | 0.829746307 |
| *PAX4* | 0 | 0 | 0 | 50 | 0 | 0 | 2.018023 | 0.01544387 | 17.06966 | 0.6646668 |
| ***PCSK9*** | **3** | **0** | **0** | **87** | **0** | **4** | **6.558343** | **1.188605** | **25.45288** | **0.03365157** |
| *ROBO2* | 0 | 0 | 0 | 83 | 0 | 3 | 0.5860782 | 0.004573575 | 4.423871 | 0.6867734932 |
| ***SOX6*** | **2** | **0** | **0** | **30** | **0** | **0** | **16.70542** | **2.816469** | **76.53032** | **0.004277757** |
| ***ST3GAL3*** | **1** | **0** | **0** | **6** | **0** | **0** | **28.86255** | **2.450073** | **252.7442** | **0.0123379** |
| *TRIM36* | 1 | 0 | 0 | 68 | 0 | 1 | 2.504773 | 0.25269 | 12.00308 | 0.3671863 |
| *TUBB3* | 0 | 0 | 0 | 6 | 0 | 0 | 5.59167 | 0.03823362 | 81.31024 | 0.3703267 |
| *TUBB4A* | 0 | 0 | 0 | 2 | 0 | 0 | 8.598562 | 0.05704608 | 160.4495 | 0.2877089 |
| *WASF3* | 0 | 0 | 0 | 46 | 0 | 0 | 1.234237 | 0.009537413 | 9.841902 | 0.88854408 |
| All | 12 | 0 | 0 | 677 | 0 | 18 | 1.870877 | 0.8974935 | 3.714163 | 0.0923 |

**Supplementary Table 4. Variants with MAF less than 0.001 identified in IUIS-listed IEI genes in the cohort of 38 ICU patients**

| **Individual** | **Ethnicity** | **Gene** | **Reference transcript^a^** | **Nucleotide change** | **Amino acid change** | **Genotype** | **Inheritance model^b^** | **Global MAF^c^** | **Population MAF^c^** | **CADD score^d^** |
| --- | --- | --- | --- | --- | --- | --- | --- | --- | --- | --- |
| P1 | Swedish | *THBD* | ENST00000377103 | c.1207A>G | p.Arg403Gly | Heterozygous | AD | 0 | 0 | 18.9 |
|  |  | *POLE* | ENST00000320574 | c.3097A>G | p.Ile1033Val | Heterozygous | AR; AD | 0 | 0 | 24.2 |
|  |  | *RANBP2* | NM_006267 | c.6098A>G | p.Lys2033Arg | Heterozygous | AD | 0.0004081 | 0.001302 | 23.6 |
| P2 | Swedish | *CSF2RA* | ENST00000432318 | c.940G>A | p.Glu314Lys | Heterozygous | XR | 0.00003584 | 0.0001914 | 13.2 |
|  |  | *ITGB2* | ENST00000302347 | c.2185C>A | p.Leu729Met | Heterozygous | AR | 0 | 0 | 15.5 |
|  |  | *CIITA* | ENST00000324288 | c.2674A>G | p.Thr892Ala | Heterozygous | AR | 0.0001523 | 0.000843 | 14.5 |
|  |  | *IRF2BP2* | ENST00000366609 | c.220G>A | p.Gly74Ser | Heterozygous | AD | 0 | 0 | 12.3 |
|  |  | *TNFAIP3* | ENST00000237289 | c.2090G>A | p.Arg697Lys | Heterozygous | AD | 0.0003036 | 0.00007736 | 22.8 |
|  |  | *LPIN2* | ENST00000261596 | c.1168+6C>T | splice variant | Heterozygous |  | 0.0002871 | 0.001034 | 1.6 |
| P3 | Kurdish | *PMS2* | ENST00000265849 | c.1004A>G | p.Asn335Ser | Heterozygous | AR | 0.000273 | - | 26.6 |
|  |  | *NOD2* | ENST00000300589 | c.743T>G | p.Leu248Arg | Heterozygous | AD | 0.0005199 | - | 26 |
|  |  | *PNP* | ENST00000361505 | c.410A>G | p.Asn137Ser | Heterozygous | AR | 0.00002121 | - | 24.1 |
|  |  | *ATM* | ENST00000278616 | c.3154-5C>T | splice variant | Heterozygous | AR; AD | 0.00003186 | - | 3.2 |
|  |  | *C8B* | ENST00000371237 | c.967C>T | p.Arg323Trp | Heterozygous | AR | 0.0005239 | - | 20.3 |
|  |  | *NOTCH1* | ENST00000277541 | c.7369C>G | p.Leu2457Val | Heterozygous | AD | 0.0005784 | - | 17.7 |
|  |  | *TIRAP* | ENST00000392678 | c.67+609G>A | splice variant | Heterozygous |  | 0.0002232 | - | 10.2 |
|  |  | *PSTPIP1* | ENST00000558012 | c.376G>A | p.Val126Ile | Heterozygous | AD | 0.000005502 | - | 12.9 |
|  |  | *PRKDC* | ENST00000338368 | c.154+863G>A | splice variant | Heterozygous | AR | 0 | - | 7.8 |
|  |  | *TRAF3* | ENST00000560371 | c.-17-5G>A | splice variant | Heterozygous |  | 0.0003999 | - | 0.1 |
|  |  | *IL36RN* | ENST00000346807 | c.164C>G | p.Ser55Cys | Heterozygous | AR | 0 | - | 15.8 |
|  |  | *IRF9* | ENST00000396864 | c.991+232G>C | splice variant | Heterozygous | AR | 0.0001124 | - | 6.1 |
|  |  | *IFNAR1* | ENST00000270139 | c.1175C>T | p.Thr392Ile | Heterozygous |  | 0.00005969 | - | 0.4 |
| P4 | Iraqi | *ATAD3A* | ENST00000378755 | c.958G>A | p.Gly320Ser | Heterozygous | AR; AD | 0.00003214 | - | 17.3 |
|  |  | *COL7A1* | ENST00000328333 | c.3856C>G | p.Gln1286Glu | Heterozygous | AR; AD | 0.000007982 | - | 13.3 |
|  |  |  |  | c.1370C>T | p.Pro457Leu | Heterozygous |  | 0.00006371 | - | 22.8 |
|  |  | *MCM4* | ENST00000262105 | c.487A>G | p.Lys163Glu | Heterozygous | AR | 0.0006894 | - | 22.9 |
|  |  | *IRF8* | ENST00000268638 | c.982T>G | p.Phe328Val | Heterozygous | AR; AD | 0.0008612 | - | 22.7 |
|  |  | *TBX21* | ENST00000177694 | c.430C>T | p.His144Tyr | Heterozygous | AR | 0.000004031 | - | 23.4 |
|  |  | *TMC8* | ENST00000318430 | c.248G>C | p.Arg83Pro | Heterozygous | AR | 0.00004041 | - | 22.5 |
|  |  | *SAMD9* | ENST00000379958 | c.550G>A | p.Glu184Lys | Heterozygous | AR; AD | 0 |  | 25.2 |
|  |  | *RSPH10B* | ENST00000404406 | c.80A>G | p.Asp27Gly | Heterozygous | AR | 0.0004559 | - | 15.4 |
|  |  | *VPS13B* | ENST00000358544 | c.11959C>G | p.Pro3987Ala | Heterozygous | AR | 0.0008732 | - | 20.2 |
|  |  | *USP18* | ENST00000215794 | c.907G>A | p.Glu303Lys | Heterozygous | AR | 0.000632 | - | 24.8 |
|  |  | *TLR3* | ENST00000296795 | c.1295A>G | p.His432Arg | Heterozygous | AR; AD | 0.000237 | - | 8.6 |
| P5 | Syrian | *BLM* | ENST00000355112 | c.2333C>G | p.Ser778Cys | Heterozygous | AR | 0.0001661 | - | 27.5 |
|  |  | *PTPRC* | ENST00000367376 | c.3668T>C | p.Ile1223Thr | Heterozygous | AR | 0.000003995 | - | 22.9 |
|  |  | *SGSM3* | ENST00000248929 | c.1697C>T | p.Pro566Leu | Heterozygous | AR | 0.00001595 | - | 28.1 |
|  |  | *LYST* | ENST00000389793 | c.10370A>G | p.Tyr3457Cys | Heterozygous | AR | 0.000003979 | - | 19 |
|  |  | *MEFV* | ENST00000219596 | c.2080A>G | p.Met694Val | Heterozygous | AR; AD | 0.0002722 | - | 10.6 |
|  |  | *SP110* | ENST00000258381 | c.272G>A | p.Arg91His | Heterozygous |  | 0.000007078 | - | 0.1 |
|  |  | *CARD11* | ENST00000396946 | c.3145-3C>T | splice variant | Heterozygous | AR; AD | 0.000606 | - | 14.3 |
|  |  | *CLEC16A* | ENST00000409790 | c.2348C>G | p.Ser783Cys | Heterozygous |  | 0 | - | 27.8 |
|  |  | *HPS6* | ENST00000299238 | c.1621C>A | p.Pro541Thr | Heterozygous | AR | 0 | - | 22.3 |
|  |  | *SASH3* | ENST00000356892 | c.466C>T | p.Pro156Ser | Hemizygous | XR | 0.00001693 | - | 9.2 |
|  |  | *SAMD9L* | ENST00000318238 | c.626C>T | p.Thr209Met | Heterozygous | AD | 0.0000177 | - | 21.8 |
|  |  | *IL6R* | ENST00000344086 | c.194C>T | p.Pro65Leu | Heterozygous | AR | 0.0004951 | - | 0.1 |
| P6 | Chilean | *IL12RB2* | ENST00000262345 | c.151T>C | p.Cys51Arg | Heterozygous |  | 0 | 0 | 23.8 |
|  |  | *DIAPH1* | ENST00000398557 | c.3152C>A | p.Ser1051Tyr | Heterozygous | AR; AD | 0 | 0 | 28.8 |
|  |  | *MRTFA* | ENST00000355630 | c.2734C>G | p.Leu912Val | Heterozygous | AR | 0.000008068 | 0.00002923 | 21 |
|  |  | *INO80* | ENST00000401393 | c.1396-8C>A | splice variant | Heterozygous |  | 0 | 0 | 3.9 |
|  |  | *ZNFX1* | ENST00000396105 | c.4255G>A | p.Val1419Met | Heterozygous | AR | 0.0003219 | 0.0009695 | 16.6 |
|  |  | *IL7R* | ENST00000303115 | c.1371A>T | p.Gln457His | Heterozygous | AR | 0 | 0 | 24.7 |
|  |  | *TBX1* | ENST00000332710 | c.163_171dup | p.Ala55Ala57dup | Heterozygous | AD | 0 | 0 | 16.2 |
| P7 | Kurdish | *POLE2* | ENST00000216367 | c.823C>A | p.Leu275Ile | Heterozygous |  | 0.0003124 | - | 25.3 |
|  |  | *HELLS* | ENST00000348459 | c.1753A>G | p.Thr585Ala | Heterozygous | AR | 0 | - | 26.1 |
|  |  | *AEBP1* | ENST00000223357 | c.3044A>G | p.Gln1015Arg | Heterozygous | AR | 0.000003986 | - | 22.3 |
|  |  | *ERBIN* | ENST00000380943 | c.1862T>C | p.Ile621Thr | Heterozygous |  | 0.0006547 | - | 24.3 |
|  |  | *HPS6* | ENST00000299238 | c.1076C>T | p.Pro359Leu | Heterozygous | AR | 0.000003991 | - | 19.7 |
|  |  | *CTNNBL1* | ENST00000361383 | c.30+771A>G | splice variant | Heterozygous | AR | 0 | - | 6.4 |
|  |  | *STK4* | ENST00000372801 | c.116+1135T>A | splice variant | Heterozygous |  | 0.0000637 | - | 17.9 |
|  |  | *PIGA* | ENST00000333590 | c.981+8G>A | splice variant | Hemizygous | XR | 0.0003075 | - | 1.8 |
|  |  | *GFI1* | ENST00000294702 | c.925-18_925-5dup | splice variant | Heterozygous | AD | 0 | - | 7 |
|  |  | *ITPKB* | ENST00000429204 | c.1078A>G | p.Arg360Gly | Heterozygous |  | 0.000008054 | - | 19.2 |
|  |  | *IRF3* | ENST00000601291 | c.1252G>A | p.Ala418Thr | Heterozygous | AD | 0.0007794 | - | 15.2 |
| P8 | Swedish | *IRAK1* | ENST00000369980 | c.1096C>T | p.Arg366Cys | Heterozygous |  | 0.0008417 | 0.002622 | 28.2 |
|  |  | *TCIRG1* | ENST00000265686 | c.2194G>A | p.Ala732Thr | Heterozygous | AR | 0.0001914 | 0.0004978 | 27.3 |
|  |  | *HCK* | ENST00000520553 | c.953-6A>C | splice variant | Heterozygous |  | 0.0001865 | 0.001572 | 0.2 |
|  |  | *CDC42* | ENST00000400259 | c.409A>G | p.Ile137Val | Heterozygous | AD | 0.00008132 | 0.0005357 | 18.8 |
| P9 | Syrian | *NCF1* | ENST00000289473 | c.579G>A | p.Trp193Ter | Heterozygous | AR | 0.0006412 | - | 35 |
|  |  | *OTULIN* | ENST00000284274 | c.857T>G | p.Leu286Arg | Heterozygous | AR | 0 | - | 31 |
|  |  | *C8A* | ENST00000361249 | c.205T>G | p.Phe69Val | Heterozygous | AR | 0.00006734 | - | 24.5 |
|  |  | *PGM3* | ENST00000513973 | c.38A>G | p.His13Arg | Heterozygous | AR | 0.00007 | - | 22.9 |
|  |  | *TCF3* | ENST00000262965 | c.23C>T | p.Ala8Val | Heterozygous | AR; AD | 0.00008529 | - | 26.7 |
|  |  | *LIG1* | ENST00000263274 | c.1670G>A | p.Arg557His | Heterozygous | AR | 0.000007952 | - | 29 |
|  |  | *MASP1* | ENST00000337774 | c.1573C>T | p.Arg525Trp | Heterozygous | AR | 0.0005692 | - | 23.1 |
|  |  | *NOS2* | ENST00000313735 | c.2404C>T | p.Arg802Cys | Heterozygous |  | 0.00002053 | - | 12.4 |
|  |  | *SMARCAL1* | ENST00000357276 | c.333C>A | p.His111Gln | Heterozygous | AR | 0 | - | 0 |
|  |  | *CARD14* | ENST00000570421 | c.1660G>A | p.Gly554Ser | Heterozygous | AD | 0.00001652 | - | 0.2 |
|  |  | *IL2RB* | ENST00000216223 | c.89-5G>A | splice variant | Heterozygous | AR | 0.00004776 | - | 10.1 |
|  |  | *POLE2* | ENST00000216367 | c.245+38T>C | splice variant | Heterozygous |  | 0.0004544 | - | 5.3 |
| P10 | Egyptian | *VPS13B* | ENST00000358544 | c.1528C>T | p.Arg510Cys | Heterozygous | AR | 0,0001168 | - | 23.8 |
|  |  | *KMT2D* | ENST00000301067 | c.952A>C | p.Lys318Gln | Heterozygous | AD | 0 | - | 25.3 |
|  |  | *NOTCH1* | ENST00000277541 | c.2849C>T | p.Ala950Val | Heterozygous | AD | 0.000004021 | - | 22.9 |
|  |  | *IL17RA* | ENST00000319363 | c.998T>C | p.Val333Ala | Heterozygous | AR | 0 | - | 24 |
|  |  | *ADAM17* | ENST00000310823 | c.2284A>T | p.Ile762Phe | Heterozygous | AR | 0.000003977 | - | 26.9 |
|  |  | *FNIP1* | ENST00000307954 | c.422A>G | p.Asn141Ser | Heterozygous | AR | 0.00001458 | - | 21.6 |
|  |  | *NFKB2* | ENST00000369966 | c.1993A>T | p.Thr665Ser | Heterozygous | AD | 0.00007507 | - | 25 |
|  |  | *TCIRG1* | ENST00000265686 | c.1630G>A | p.Val544Met | Heterozygous | AR | 0.0001203 | - | 14.3 |
|  |  |  |  | c.-5+666A>C | splice variant | Heterozygous |  | 0.00007987 | - | 6.5 |
|  |  | *LCP2* | ENST00000046794 | c.866C>T | p.Pro289Leu | Heterozygous | AR | 0.0009411 | - | 17 |
|  |  | *GFI1* | ENST00000294702 | c.925-18_925-5dup | splice variant | Homozygous | AD | 0 | - | 7 |
| P11 | Thai | *NOTCH1* | ENST00000277541 | c.3121G>A | p.Gly1041Ser | Heterozygous | AD | 0.00002705 | 0 | 23.7 |
|  |  | *RTEL1* | ENST00000370018 | c.704G>T | p.Arg235Leu | Heterozygous | AR; AD | 0 | 0 | 24.4 |
|  |  | *SMARCAL1* | ENST00000357276 | c.2534T>A | p.Leu845Gln | Heterozygous | AR | 0.00008133 | 0.0004853 | 25.2 |
|  |  | *POLR3A* | ENST00000372371 | c.2306G>A | p.Arg769Gln | Heterozygous | AR | 0.00006405 | 0.00007362 | 22.8 |
|  |  | *C1S* | ENST00000360817 | c.1388C>T | p.Ala463Val | Heterozygous | AD | 0.00001193 | 0.00006933 | 22.5 |
|  |  | *DNAJC21* | ENST00000382021 | c.622G>T | p.Ala208Ser | Heterozygous | AR | 0 | 0 | 22.8 |
|  |  | *KMT2D* | ENST00000301067 | c.10467G>T | p.Gln3489His | Heterozygous | AD | 0.00007638 | 0.0001427 | 22.2 |
|  |  | *MRE11* | ENST00000323929 | c.352A>T | p.Ile118Phe | Heterozygous | AR | 0 | 0 | 25.1 |
|  |  | *FASLG* | ENST00000367721 | c.451+8T>A | splice variant | Heterozygous | AD | 0.00003536 | 0.0005546 | 11.5 |
|  |  | *TIRAP* | ENST00000392678 | c.382G>A | p.Ala128Thr | Heterozygous |  | 0.00001614 | 0 | 24.4 |
|  |  | *EPG5* | ENST00000282041 | c.3391A>G | p.Ile1131Val | Heterozygous | AR | 0.0008244 | 0.009582 | 14.1 |
|  |  | *VPS13B* | ENST00000358544 | c.10943-7G>A | splice variant | Heterozygous | AR | 0.00007072 | 0.0003466 | 0 |
|  |  | *IL10RA* | NM_001558 | c.1693C>A | p.Leu565Met | Heterozygous | AR | 0.0001044 | 0.001111 | 17.3 |
|  |  | *TRNT1* | ENST00000251607 | c.810_811insAAACTT | p.Pro270_Ala271insLysLeu | Heterozygous | AR | 0.001248 | 0.0143 | 22.3 |
| P12 | Iraqi | *RTEL1* | ENST00000370018 | c.680A>G | p.Asn227Ser | Heterozygous | AR; AD | 0.00002784 | - | 25.7 |
|  |  | *TCIRG1* | ENST00000265686 | c.148C>T | p.Arg50Cys | Homozygous | AR | 0.0000508 | - | 29.4 |
|  |  | *NPC1* | ENST00000269228 | c.2141G>A | p.Arg714His | Heterozygous | AR | 0.0001255 | - | 27 |
|  |  | *PAX1* | ENST00000398485 | c.956A>G | p.Lys319Arg | Heterozygous | AR | 0 | - | 25.1 |
|  |  | *NOS2* | ENST00000313735 | c.272G>A | p.Gly91Asp | Heterozygous |  | 0 | - | 22.2 |
|  |  | *ATAD3A* | ENST00000378755 | c.1876G>A | p.Gly626Arg | Heterozygous | AR; AD | 0.0005993 | - | 4.6 |
|  |  | *LRBA* | ENST00000357115 | c.3143T>C | p.Leu1048Ser | Heterozygous | AR | 0 | - | 1.9 |
|  |  | *TCF3* | ENST00000262965 | c.307G>A | p.Gly103Ser | Heterozygous | AR; AD | 0.0003321 | - | 0 |
|  |  | *FERMT1* | ENST00000217289 | c.1594-6G>A | splice variant | Heterozygous | AR | 0.0002958 | - | 0.7 |
|  |  | *ARPC1B* | ENST00000252725 | c.718C>G | p.Leu240Val | Heterozygous | AR | 0.00001195 | - | 21.3 |
|  |  | *AIRE* | ENST00000291582 | c.996-7C>T | splice variant | Heterozygous | AR; AD | 0.0002874 | - | 0.2 |
| P13 | Romani | *USP43* | ENST00000285199 | c.1360G>A | p.Gly454Arg | Heterozygous |  | 0.0008968 | - | 19.5 |
|  |  | *FCGR3B* | ENST00000294800 | c.41-3C>T | splice variant | Heterozygous | AR | 0.0001079 | - | 14.5 |
|  |  | *NCKAP1L* | ENST00000293373 | c.2698+7A>G | splice variant | Heterozygous | AR | 0.0003116 | - | 0.1 |
|  |  | *COL7A1* | ENST00000328333 | c.5339G>A | p.Arg1780Gln | Heterozygous | AR; AD | 0.00003895 | - | 29.2 |
|  |  |  |  | c.2791C>T | p.Arg931Cys | Heterozygous |  | 0.0002463 | - | 23.5 |
|  |  | *DOCK8* | ENST00000432829 | c.2840G>A | p.Arg947His | Heterozygous | AR | 0.00003978 | - | 25.8 |
|  |  | *VPS45* | ENST00000369128 | c.443C>T | p.Pro148Leu | Heterozygous | AR | 0.000413 | - | 28.7 |
|  |  | *CFHR3* | ENST00000367425 | c.914G>A | p.Gly305Glu | Heterozygous | AR; AD | 0.00005451 | - | 22.8 |
| P14 | Central American | *SNX10* | ENST00000338523 | c.25-1G>C | splice variant | Heterozygous | AR | 0 | - | 33 |
|  |  | *SKIC2* | ENST00000375394 | c.1846C>T | p.Arg616Cys | Heterozygous | AR | 0.00002527 | - | 32 |
|  |  | *INO80* | ENST00000401393 | c.4163C>G | p.Ala1388Gly | Heterozygous |  | 0.00001193 | - | 22.6 |
|  |  | *STK4* | ENST00000372806 | c.1172C>T | p.Ala391Val | Heterozygous |  | 0.0001216 | - | 21.6 |
|  |  | *NOTCH1* | ENST00000277541 | c.608G>A | p.Arg203His | Heterozygous | AD | 0.0002458 | - | 16.5 |
|  |  | *TYK2* | ENST00000525621 | c.1153C>T | p.His385Tyr | Heterozygous | AR | 0 | - | 26.1 |
|  |  | *IL7R* | ENST00000303115 | c.590C>T | p.Pro197Leu | Heterozygous | AR | 0.00002791 | - | 17.6 |
|  |  | *SLC29A3* | ENST00000373189 | c.1133C>T | p.Ala378Val | Heterozygous | AR | 0.0001733 | - | 13.9 |
|  |  | *SAMHD1* | ENST00000262878 | c.208+8C>T | splice variant | Heterozygous | AR; AD | 0.0001982 | - | 12.1 |
|  |  | *TMC6* | ENST00000322933 | c.154G>C | p.Ala52Pro | Heterozygous | AR | 0.0007032 | - | 23.9 |
|  |  | *CR2* | ENST00000367058 | c.2989G>A | p.Val997Met | Heterozygous | AR | 0.00002389 | - | 11.3 |
|  |  | *USP18* | ENST00000215794 | c.970C>T | p.Arg324Trp | Heterozygous | AR | 0.00005993 | - | 17.6 |
|  |  | *DCLRE1C* | ENST00000378246 | c.1388C>T | p.Tyr463Cys | Heterozygous | AR | 0.0001662 | - | 1.2 |
|  |  | *MALT1* | ENST00000348428 | c.391G>T | p.Val131Leu | Heterozygous | AR | 0 | - | 15.5 |
|  |  | *NLRC4* | ENST00000360906 | c.1003A>C | p.Met335Leu | Heterozygous | AD | 0 | - | 18.2 |
| P15 | Mongolian | *TTC7A* | ENST00000319190 | c.1790C>A | p.Pro597His | Heterozygous | AR | 0 | - | 29 |
|  |  | *MYO5A* | ENST00000399231 | c.2479A>T | p.Met827Leu | Heterozygous | AR | 0 | - | 24 |
|  |  | *PIGA* | NM_002641 | c.1188+4T>C | splice variant | Heterozygous | XR | 0.00001657 | - | 11.8 |
|  |  | *SKIC3* | ENST00000358746 | c.964C>T | p.Pro322Ser | Heterozygous | AR | 0.0005486 | - | 19.8 |
|  |  | *MEFV* | ENST00000219596 | c.761_764dup | p.Asn256ArgfsX70 | Heterozygous | AR; AD | 0.000007955 | - | 20 |
|  |  | *TCF3* | ENST00000262965 | c.1162G>A | p.Gly388Ser | Heterozygous | AR; AD | 0.000006238 | - | 17.5 |
|  |  | *SEMA3E* | ENST00000307792 | c.2258A>G | p.Asn753Ser | Heterozygous | AD | 0 | - | 13.8 |
|  |  | *MRE11* | ENST00000323929 | c.1780A>G | p.Arg594Gly | Heterozygous | AR | 0.0009652 | - | 23.1 |
|  |  | *SLC46A1* | ENST00000440501 | c.370C>G | p.Leu124Val | Heterozygous | AR | 0.00002427 | - | 23.1 |
|  |  | *ICOSLG* | ENST00000400379 | c.452A>G | p.Gln151Arg | Heterozygous | AR | 0 | - | 12.7 |
| P16 | Kenyan | *NRAS* | ENST00000369535 | c.*5-1G>A | splice variant | Heterozygous | AD | 0 | 0 | 33 |
|  |  | *ERBIN* | ENST00000380943 | c.910C>G | p.Leu304Val | Heterozygous |  | 0.0002533 | 0.002501 | 25.5 |
|  |  | *CSF2RB* | ENST00000262825 | c.718+4A>T | splice variant | Heterozygous | AR | 0 | 0 | 10.8 |
|  |  |  |  | c.2506C>T | p.Pro836Ser | Heterozygous |  | 0 | 0 | 0 |
|  |  | *TIRAP* | ENST00000392678 | c.651G>A | splice variant | Heterozygous |  | 0.00000401 | 0.00006458 | 19.4 |
|  |  | *UNC93B1* | ENST00000227471 | c.610A>C | p.Met204Leu | Heterozygous |  | 0.000008038 | 0.0001301 | 10.1 |
|  |  |  |  | c.1774G>C | p.Gly592Arg | Heterozygous |  | 0.0003254 | 0.001093 | 17.4 |
|  |  |  |  | c.1778C>T | p.Pro593Leu | Heterozygous |  | 0 | 0 | 15.7 |
|  |  | *IL18BP* | ENST00000404792 | c.29-4T>A | splice variant | Heterozygous | AR | 0.00003627 | 0.0003305 | 2.6 |
|  |  | *NLRP1* | ENST00000345221 | c.2841G>C | p.Arg947Ser | Heterozygous | AR; AD | 0.0002343 | 0.002445 | 16.2 |
|  |  | *RELA* | ENST00000406246 | c.619C>T | p.Leu207Phe | Heterozygous | AD | 0 | 0 | 23.5 |
|  |  | *TNFRSF18* | ENST00000379268 | c.175C>T | p.Arg59Cys | Heterozygous | AR | 0.000006996 | 0.0000442 | 14.9 |
|  |  | *ACAA1* | ENST00000333167 | c.225C>G | p.Asp75Glu | Heterozygous | AR | 0.0004181 | 0.00297 | 14.2 |
|  |  | *TFRC* | ENST00000360110 | c.1791C>G | p.Phe597Leu | Heterozygous | AR | 0.000003976 | 0.00006152 | 7.7 |
|  |  | *DIAPH1* | ENST00000398557 | c.300+4A>T | splice variant | Heterozygous | AR; AD | 0 | 0 | 21.5 |
|  |  | *MASP2* | ENST00000400897 | c.2053G>A | p.Asp685Asn | Heterozygous | AR | 0.0003515 | 0.00256 | 9.5 |
|  |  | *TLR2* | ENST00000260010 | c.1906A>C | p.Ser636Arg | Heterozygous | AD | 0.0003292 | 0.003329 | 0 |
|  |  | *IL6R* | ENST00000368485 | c.1144A>C | p.Ile382Leu | Heterozygous | AR | 0.000003979 | 0.00003269 | 6.4 |
|  |  | *POLD1* | ENST00000440232 | c.3068-6C>G | splice variant | Heterozygous | AD | 0.000006452 | 0.00004048 | 8.9 |
|  |  | *IL2RB* | ENST00000216223 | c.459A>C | p.Glu153Asp | Heterozygous | AR | 0.000297 | 0.003205 | 0 |
|  |  | *IL17RC* | ENST00000383812 | c.465+7A>G | splice variant | Heterozygous | AR | 0 | 0 | 8.6 |
|  |  | *PTPRC* | ENST00000367376 | c.3140T>C | p.Met1047Thr | Heterozygous | AR | 0 | 0 | 26.6 |
|  |  | *DOCK2* | ENST00000256935 | c.3140T>G | p.Phe1047Cys | Heterozygous | AR | 0.00006015 | 0.0004005 | 31 |
|  |  | *NLRP12* | ENST00000324134 | c.850C>T | p.Arg284* | Heterozygous | AD | 0.0001487 | 0.001365 | 33 |
| P17 | Chilean | *PTPRC* | ENST00000367376 | c.3124G>A | p.Gly1042Ser | Heterozygous | AR | 0.000007971 | 0.00005799 | 12.5 |
|  |  | *TNFRSF4* | ENST00000379236 | c.419C>T | p.Ala140Val | Heterozygous | AR | 0 | 0 | 21.7 |
|  |  | *NOTCH1* | ENST00000277541 | c.6083-5C>T | splice variant | Heterozygous | AD | 0.00007232 | 0.00008692 | 1.6 |
|  |  | *IL10RB* | ENST00000290200 | c.871G>A | p.Val29Ile | Heterozygous | AR | 0 | 0 | 22.3 |
|  |  | *JAK1* | ENST00000342505 | c.1978G>A | p.Asp660Asn | Heterozygous | AD | 0.0002138 | 0.001387 | 23.2 |
|  |  | *CARD14* | ENST00000570421 | c.299A>G | p.Tyr100Cys | Heterozygous | AD | 0.00004375 | 0.0002892 | 23.8 |
|  |  | *AIRE* | ENST00000291582 | c.880-203G>C | splice variant | Heterozygous | AR; AD | 0.000003989 | 0.000008836 | 1.7 |
|  |  | *MAN2B2* | ENST00000285599 | c.86C>T | p.Ala29Val | Heterozygous |  | 0.0005662 | 0.002241 | 13.3 |
|  |  | *CTC1* | ENST00000315684 | c.1573C>T | p.His525Tyr | Heterozygous | AR | 0 | 0 | 0.4 |
| P18 | Turkish | *NOTCH1* | ENST00000277541 | c.3853G>A | p.Val1285Met | Heterozygous | AD | 0.00005234 | - | 26.7 |
|  |  | *RORC* | ENST00000356728 | c.190C>T | p.His64Tyr | Heterozygous | AR | 0.0002849 | - | 25.9 |
|  |  | *CSF2RB* | ENST00000262825 | c.1795T>G | p.Tyr599Asp | Heterozygous | AR | 0 | - | 24.7 |
|  |  | *TCF3* | ENST00000262965 | c.878T>C | p.Phe293Ser | Heterozygous | AR; AD | 0.0001362 | - | 24.9 |
|  |  | *LIG4* | ENST00000356922 | c.2639G>T | p.Arg880Ile | Heterozygous | AR | 0 | - | 25.3 |
|  |  | *MEFV* | ENST00000219596 | c.1016C>T | p.Ser339Phe | Heterozygous | AR; AD | 0.0001789 | - | 16.7 |
|  |  | *TNFRSF13B* | ENST00000261652 | c.204dup | p.Leu69ThrfsX12 | Heterozygous | AR; AD | 0.0004002 | - | 28.2 |
|  |  | *MTHFD1* | ENST00000216605 | c.2077C>T | p.His693Tyr | Heterozygous | AR | 0.000003977 | - | 25.1 |
|  |  | *C3* | ENST00000245907 | c.1027C>T | p.Arg343Cys | Heterozygous | AR; AD | 0.00004598 | - | 20.6 |
|  |  | *ITPKB* | ENST00000429204 | c.739G>C | p.Glu247Gln | Heterozygous |  | 0.00008539 | - | 12.8 |
|  |  | *NLRP1* | ENST00000345221 | c.2290G>C | p.Val764Leu | Heterozygous | AR; AD | 0.00002386 | - | 13.6 |
|  |  | *TERT* | ENST00000310581 | c.769G>T | p.Ala257Ser | Heterozygous | AR; AD | 0.000005316 | - | 2.1 |
|  |  | *IL17RA* | ENST00000319363 | c.310+3G>A | splice variant | Heterozygous | AR | 0.00002478 | - | 13.3 |
|  |  | *TYK2* | ENST00000525621 | c.1576G>A | p.Val526Ile | Heterozygous | AR | 0.0001489 | - | 6.5 |
| P19 | Eritrean | *ATM* | ENST00000278616 | c.2426C>T | p.Ser809Leu | Heterozygous | AR; AD | 0 | 0 | 25.5 |
|  |  |  |  | c.5185G>C | p.Val1729Leu | Heterozygous |  | 0.0001026 | 0.00004006 | 23.1 |
|  |  | *EXTL3* | ENST00000220562 | c.803C>T | p.Thr268Met | Heterozygous | AR | 0.0001874 | 0.0001202 | 24.5 |
|  |  | *STAT2* | ENST00000557235 | c.1454C>T | p.Pro485Leu | Heterozygous | AR | 0.0002348 | 0.00004072 | 18.8 |
|  |  | *LRBA* | ENST00000510413 | c.4124T>G | p.Ile1375Arg | Heterozygous | AR | 0 | 0 | 27.8 |
|  |  | *ACTB* | ENST00000331789 | c.6-135C>T | splice variant | Heterozygous | AD | 0.000006377 | 0 | 17.6 |
|  |  | *FAS* | ENST00000355740 | c.236A>C | p.Glu79Ala | Heterozygous | AD | 0 | 0 | 4.2 |
|  |  | *ERLIN1* | ENST00000421367 | c.-8G>T | splice variant | Heterozygous | AR | 0 | 0 | 16.6 |
|  |  | *IRAK1* | ENST00000369980 | c.1106G>A | p.Gly369Glu | Hemizygous | XR | 0.000137 | 0 | 22.5 |
|  |  | *STAT2* | ENST00000557235 | c.250C>A | p.Gln84Lys | Heterozygous | AR | 0.0002228 | 0 | 1.2 |
|  |  | *SHARPIN* | ENST00000398712 | c.492G>C | p.Arg164Ser | Heterozygous | AR | 0.00006407 | 0.0001155 | 11.1 |
|  |  | *FCHO1* | ENST00000596536 | c.2401G>A | p.Val801Ile | Heterozygous | AR | 0.0001393 | 0.001414 | 15.8 |
|  |  | *NLRP1* | ENST00000345221 | c.822T>G | p.Phe274Leu | Heterozygous | AR; AD | 0.0005762 | 0.001322 | 0.3 |
|  |  |  |  | c.736A>G | p.Thr246Ala | Heterozygous |  | 0.000581 | 0.001332 | 0 |
|  |  | *APOL1* | ENST00000319136 | c.980C>T | p.Ser327Leu | Heterozygous |  | 0.00004243 | 0.0002003 | 19.5 |
| P20 | Somalian | *PSTPIP1* | ENST00000558012 | c.700C>T | p.His234Tyr | Heterozygous | AD | 0 | 0 | 25.1 |
|  |  | *COL7A1* | ENST00000328333 | c.1507+1G>C | splice variant | Heterozygous | AR; AD | 0 | 0 | 35 |
|  |  | *NFKB2* | ENST00000369966 | c.1993A>T | p.Thr665Ser | Heterozygous | AD | 0.00007507 | 0 | 25 |
|  |  | *INO80* | ENST00000401393 | c.2586G>T | p.Arg862Ser | Heterozygous |  | 0 | 0 | 20.1 |
|  |  | *SKIC3* | ENST00000358746 | c.1187A>T | p.Tyr396Phe | Heterozygous | AR | 0 | 0.00003185 | 22.6 |
|  |  | *VPS13B* | ENST00000358544 | c.11821-5C>A | splice variant | Heterozygous | AR | 0 | 0 | 12.1 |
|  |  | *CYC1* | ENST00000318911 | c.323C>A | p.Thr108Asn | Heterozygous | AR | 0.0001739 | 0.00161 | 23.5 |
|  |  |  |  | c.35T>C | p.Val12Ala | Heterozygous |  | 0 | 0.00006526 | 15.5 |
|  |  | *RTEL1* | ENST00000370018 | c.3463G>A | p.Val1179Met | Heterozygous | AR; AD | 0.0001519 | 0.000367 | 0.1 |
|  |  | *STAT2* | ENST00000557235 | c.1020A>G | splice variant | Heterozygous | AR | 0.00003981 | 0 | 14.5 |
|  |  | *HYOU1* | ENST00000404233 | c.795-3C>T | splice variant | Heterozygous | AR | 0.000008168 | 0.00006256 | 13.2 |
|  |  | *LCP2* | ENST00000046794 | c.1100+3T>C | splice variant | Heterozygous | AR | 0.0005275 | 0.00004133 | 8.2 |
|  |  | *ELANE* | ENST00000263621 | c.23C>G | p.Ala8Gly | Heterozygous | AD | 0 | 0 | 10.5 |
|  |  | *IKBKB* | ENST00000519735 |  | p.Trp254* | Heterozygous | AR; AD | 0,0008 | 0,0002 | 2.6 |
|  |  | *CD8A* | ENST00000283635 | c.626-5T>C | splice variant | Heterozygous | AR | 0 | 0.00000399 | 9.9 |
| P21 | Turkish | *RELB* | ENST00000221452 | c.491T>G | p.Leu164Arg | Heterozygous | AR | 0 | - | 25.6 |
|  |  | *TNFRSF1A* | ENST00000162749 | c.434A>G | p.Asn145Ser | Heterozygous | AD | 0.00009178 | - | 33 |
|  |  | *IFNGR1* | ENST00000367739 | c.589G>A | p.Glu197Lys | Heterozygous | AR; AD | 0.0002123 | - | 21.2 |
|  |  | *SGSM3* | ENST00000248929 | c.1629+4A>G | splice variant | Heterozygous | AR | 0.0001675 | - | 15.2 |
|  |  | *ICOS* | ENST00000316386 | c.59-3C>T | splice variant | Heterozygous | AR | 0.00001603 | - | 18.2 |
| P22 | Syrian | *TYK2* | ENST00000525621 | c.1342C>T | p.Arg448Trp | Heterozygous | AR | 0.0001032 | - | 23.5 |
|  |  | *ATM* | ENST00000278616 | c.3382C>G | p.Gln1128Glu | Heterozygous | AR; AD | 0.000003985 | - | 15.8 |
|  |  | *SH3KBP1* | ENST00000397821 | c.1840A>G | p.Thr614Ala | Hemizygous | XR | 0 | - | 20.2 |
|  |  | *DEF6* | ENST00000316637 | c.1228A>C | p.Met410Leu | Heterozygous | AR | 0 | - | 27.2 |
|  |  | *PRKDC* | ENST00000338368 | c.8659G>C | p.Val2887Leu | Heterozygous | AR | 0.000798 | - | 14.9 |
|  |  | *PIK3CD* | ENST00000377346 | c.58G>A | p.Val20Ile | Heterozygous | AR; AD | 0.00004595 | - | 15.8 |
|  |  | *IRF7* | ENST00000397574 | c.1122C>T | splice variant | Heterozygous | AR | 0.00004726 | - | 12.4 |
| P23 | Unknown | *NLRP3* | ENST00000336119 | c.860T>A | p.Ile287Asn | Heterozygous | AD | 0 | - | 21.6 |
|  |  | *RAC2* | ENST00000249071 | c.281G>A | p.Arg94His | Heterozygous | AR; AD | 0.00001908 | - | 23.4 |
| P24 | Egyptian | *COL7A1* | ENST00000328333 | c.2545G>A | p.Gly849Arg | Heterozygous | AR; AD | 0.00004244 | - | 28.5 |
|  |  | *SLC7A7* | ENST00000397532 | c.740A>G | p.Tyr247Cys | Heterozygous | AR | 0.000007954 | - | 27.2 |
|  |  | *ELANE* | ENST00000263621 | c.271C>T | p.Arg91Trp | Heterozygous | AD | 0.00000423 | - | 22.5 |
|  |  | *ARPC1B* | ENST00000252725 | c.639C>G | p.Ser213Arg | Heterozygous | AR | 0 | - | 19.9 |
|  |  | *ATM* | ENST00000278616 | c.1492G>C | p.Glu498Gln | Heterozygous | AR; AD | 0 | - | 17.6 |
|  |  | *MAN2B2* | ENST00000285599 | c.112G>A | p.Asp38Asn | Heterozygous |  | 0.0002687 | - | 26.2 |
|  |  | *DOCK2* | ENST00000256935 | c.2702C>T | p.Ala901Val | Heterozygous | AR | 0.00001602 | - | 9.7 |
|  |  | *KMT2D* | ENST00000301067 | c.2409A>C | p.Glu803Asp | Heterozygous | AD | 0 | - | 12.8 |
|  |  | *ADAR* | ENST00000368474 | c.1910A>G | p.Lys637Aeg | Heterozygous | AR; AD | 0 | - | 19.9 |
| P25 | Bosnian | *DOCK8* | ENST00000432829 | c.812C>T | p.Pro271Leu | Heterozygous | AR | 0.0001416 | 0.0002248 | 26.2 |
|  |  | *COL7A1* | ENST00000328333 | c.8017C>T | p.Pro2673Ser | Heterozygous | AR; AD | 0.00008371 | 0.00005294 | 25.9 |
|  |  | *PMS2* | ENST00000265849 | c.2324A>G | p.Asn775Ser | Heterozygous | AR | 0.0003278 | 0.0004929 | 23.1 |
|  |  | *KLF1* | ENST00000264834 | c.803G>T | p.Arg268Leu | Heterozygous | AR | 0.0001388 | 0.0001812 | 27.1 |
|  |  | *LIG4* | ENST00000356922 | c.381G>C | p.Met127Ile | Heterozygous | AR | 0.0000637 | 0.00007754 | 11.6 |
|  |  |  |  | c.1909G>A | p.Val637Ile | Heterozygous |  | 0 | 0 | 10.1 |
|  |  | *IRF2BP2* | ENST00000366609 | c.968G>A | p.Ser323Asn | Heterozygous | AD | 0.0001165 | 0.00009608 | 20.8 |
|  |  | *ICOSLG* | ENST00000400379 | c.910del | p.Val304TrpfsX250 | Heterozygous | AR | 0.0007163 | 0.001452 | 0.1 |
|  |  | *CYBA* | ENST00000261623 | c.223G>A | p.Ala75Thr | Heterozygous | AR | 0.00004607 | 0.000007767 | 0.1 |
|  |  | *IL12RB1* | ENST00000600835 | c.1879G>A | p.Glu627Lys | Heterozygous | AR | 0.0008422 | 0.0008562 | 14.5 |
|  |  | *RC3H1* | ENST00000258349 | c.3202C>T | p.Pro1068Ser | Heterozygous |  | 0.00002386 | 0.00004396 | 21.4 |
| P26 | South Korean | *CD247* | ENST00000392122 | c.391C>T | p.Arg131Cys | Heterozygous | AR | 0.00005172 | 0.0005238 | 26 |
|  |  | *CLEC16A* | ENST00000409790 | c.2869G>A | p.Glu957Lys | Heterozygous |  | 0.0001461 | 0.0002619 | 20.3 |
|  |  | *IKBKG* | ENST00000369601 | c.549G>C | p.Gln183His | Heterozygous | XR, XD | 0 | 0 | 22.3 |
|  |  | *CHD7* | ENST00000423902 | c.7145C>T | p.Thr2382Met | Heterozygous | AD | 0.00002429 | 0 | 20.6 |
|  |  | *RAG1* | ENST00000299440 | c.2554A>T | p.Met852Leu | Heterozygous | AR | 0.00004957 | 0.001835 | 25.4 |
|  |  | *STAT5B* | ENST00000293328 | c.389C>T | p.Ala130Val | Heterozygous | AR; AD | 0.000205 | 0.009167 | 11.6 |
|  |  | *GIMAP6* | ENST00000328902 | c.433C>T | p.Arg145Cys | Heterozygous |  | 0.00008773 | 0.0002626 | 13.9 |
|  |  | *IL23R* | ENST00000347310 | c.662C>T | p.Ser221Phe | Heterozygous |  | 0.0001023 | 0.0043 | 27 |
|  |  | *IL6R* | ENST00000368485 | c.335T>G | p.Val112Gly | Heterozygous | AR | 0 | 0 | 11.3 |
|  |  | *ATAD3A* | ENST00000378755 | c.724C>T | p.Arg242Trp | Heterozygous | AR | 0.0008785 | 0.01285 | 19.6 |
|  |  | *CSF2RB* | ENST00000262825 | c.1587-5C>T | splice variant | Heterozygous | AR | 0.0001282 | 0.006024 | 5.3 |
|  |  | *GIMAP5* | ENST00000358647 | c.850G>A | p.Glu284Lys | Heterozygous | AR | 0.00005587 | 0.002619 | 0 |
|  |  | *HPS1* | ENST00000325103 | c.952C>G | p.Leu318Val | Heterozygous | AR | 0.0009432 | 0.01134 | 17.5 |
|  |  | *CFH* | ENST00000367429 | c.3178G>C | p.Val1060Leu | Heterozygous | AR; AD | 0.0005801 | 0.01298 | 0 |
|  |  |  |  | c.3172T>C | p.Tyr1058His | Heterozygous |  | 0.0006296 | 0.01376 | 0 |
|  |  | *RHOH* | ENST00000381799 | c.263A>G | p.His88Arg | Heterozygous | AR | 0.00003581 | 0 | 20 |
|  |  | *PSTPIP1* | ENST00000558012 | c.563-8G>A | splice variant | Heterozygous | AD | 0.0007683 | 0.009434 | 3.7 |
|  |  | *SKIC3* | ENST00000358746 | c.4153G>A | p.Val1385Ile | Heterozygous | AR | 0.000792 | 0.02043 | 21.8 |
|  |  | *SAMD9L* | ENST00000318238 | c.981A>G | splice variant | Heterozygous | AD | 0 | 0 | 11.2 |
|  |  | *HYOU1* | ENST00000404233 | c.1125T>G | p.Asp375Glu | Heterozygous | AR | 0 | 0 | 20.4 |
|  |  | *TCIRG1* | ENST00000265686 | c.714-20G>A | splice variant | Heterozygous | AR | 0.000834 | 0.008071 | 3.8 |
| P27 | Iranian | *IL23R* | ENST00000347310 | c.658C>A | p.Pro220Thr | Heterozygous | AR | 0 | - | 25.5 |
|  |  | *SLCO2A1* | ENST00000310926 | c.1808G>A | p.Arg603Gln | Heterozygous | AR; AD | 0.00002878 | - | 25 |
|  |  | *RASGRP1* | ENST00000310803 | c.1643C>G | p.Thr548Ser | Heterozygous | AR | 0 | - | 24.3 |
|  |  | *GIMAP6* | ENST00000328902 | c.625G>C | p.Glu209Gln | Heterozygous |  | 0 | - | 22.4 |
|  |  | *IL23R* | ENST00000347310 | c.664G>T | p.Ala222Ser | Heterozygous |  | 0 | - | 18.4 |
|  |  | *PIK3CD* | ENST00000377346 | c.1021-7T>A | splice variant | Heterozygous | AR; AD | 0 | - | 9.8 |
|  |  | *LCP2* | ENST00000046794 | c.1100+3T>C | splice variant | Heterozygous | AR | 0.0005275 | - | 8.2 |
|  |  | *CIITA* | ENST00000324288 | c.3004G>A | p.Glu1002Lys | Heterozygous | AR | 0.00003594 | - | 20.7 |
|  |  | *SOCS1* | ENST00000332029 | c.106C>G | p.Pro36Ala | Heterozygous | AD | 0 | - | 12.4 |
|  |  | *SMARCD2* | ENST00000448276 | c.134C>T | p.Pro45Leu | Heterozygous | AR | 0.00003328 | - | 24.7 |
|  |  | *IL23R* | ENST00000347310 | c.653-4C>A | splice variant | Heterozygous | AR | 0 | - | 9 |
|  |  | *FCHO1* | ENST00000596536 | c.1129G>A | p.Ala377Thr | Heterozygous | AR | 0.0001641 | - | 15.7 |
| P28 | Thai | *G6PD* | ENST00000393562 | c.567G>C | p.Met189Ile | Hemizygous | XR | 0.0008693 | 0 | 22.1 |
|  |  | *COPG1* | ENST00000314797 | c.812C>T | p.Ala271Val | Heterozygous |  | 0.00001194 | 0.000208 | 22.8 |
|  |  | *IRF8* | ENST00000268638 | c.600A>G | splice variant | Heterozygous | AR; AD | 0.000007965 | 0.00006935 | 20.3 |
|  |  | *DCLRE1C* | ENST00000378246 | c.334-3C>T | splice variant | Heterozygous | AR | 0.00007779 | 0.001109 | 2.2 |
|  |  | *LPIN2* | ENST00000261596 | c.2655C>A | p.Asp885Glu | Heterozygous |  | 0.0000283 | 0.00006937 | 14.6 |
|  |  | *COL7A1* | ENST00000328333 | c.3107G>A | p.Arg1036Gln | Heterozygous | AR; AD | 0.00006406 | 0 | 0 |
|  |  | *LCP2* | ENST00000046794 | c.610C>T | p.Arg204Trp | Heterozygous | AR | 0 | 0 | 22.4 |
|  |  | *CSF3R* | ENST00000373103 | c.1725C>T | splice variant | Heterozygous | AR; AD | 0.000003981 | 0 | 9 |
|  |  | *DNASE2* | ENST00000222219 | c.620G>A | p.Ser207Asn | Heterozygous | AR; AD | 0.000003976 | 0 | 0.1 |
| P29 | Kurdish | *DOCK2* | ENST00000256935 | c.847C>T | p.Leu283Phe | Heterozygous | AR | 0 | - | 26.1 |
|  |  |  |  | c.1603A>G | p.Met535Val | Heterozygous |  | 0.0004139 | - | 25.4 |
|  |  | *MCM10* | ENST00000378714 | c.1280C>A | p.Ala427Glu | Heterozygous | AR | 0.00002477 | - | 23.3 |
|  |  | *CRACR2A* | ENST00000252322 | c.56C>A | p.Ser19Tyr | Heterozygous |  | 0 | - | 10.7 |
|  |  | *PRKDC* | ENST00000338368 | c.11602C>G | p.Leu3868Val | Heterozygous | AR | 0.0006006 | - | 21.2 |
|  |  | *CFP* | ENST00000247153 | c.121G>A | p.Gly41Ser | Hemizygous | XR | 0.0002336 | - | 13.9 |
|  |  | *PIGA* | ENST00000333590 | c.981+8G>A | splice variant | Hemizygous | XR | 0.0003075 | - | 1.8 |
|  |  | *IFNAR2* | ENST00000342136 | c.884C>T | p.Pro295Leu | Heterozygous | AR | 0.00008954 | - | 9.3 |
| P30 | Turkish | *NLRP3* | ENST00000336119 | c.860T>A | p.Ile287Asn | Heterozygous | AD | 0 | - | 21.6 |
|  |  | *SERPING1* | ENST00000278407 | c.292C>A | p.Pro98Thr | Heterozygous | AR; AD | 0 | - | 11.2 |
|  |  | *STXBP2* | ENST00000221283 | c.1459G>T | p.Val487Leu | Heterozygous | AR | 0.00000753 | - | 19.5 |
|  |  | *IRAK1* | ENST00000369980 | c.1106G>A | p.Gly369Glu | Hemizygous |  | 0.000137 | - | 22.5 |
|  |  | *TRAF3* | ENST00000560371 | c.1099G>A | p.Val367Met | Heterozygous |  | 0.00008495 | - | 22.2 |
|  |  | *SENP3* | ENST00000321337 | c.713C>G | p.Ser238Trp | Heterozygous |  | 0 | - | 29.6 |
|  |  | *LCP2* | ENST00000046794 | c.1100+3T>C | splice variant | Homozygous | AR | 0.0005275 | - | 8.2 |
|  |  | *HELLS* | ENST00000348459 | c.1770C>T | splice variant | Heterozygous | AR | 0.00003231 | - | 11.3 |
|  |  | *IL12RB2* | ENST00000262345 | c.1423C>T | p.Arg475Trp | Heterozygous |  | 0.0002547 | - | 16.3 |
|  |  | *IL17RA* | ENST00000319363 | c.310+3G>A | splice variant | Heterozygous | AR | 0.00002478 | - | 13.3 |
| P31 | Balkan | *COL7A1* | ENST00000328333 | c.5086C>T | p.Arg1696Cys | Heterozygous | AR; AD | 0.000212 | - | 32 |
|  |  | *PMS2* | ENST00000265849 | c.857A>G | p.Asp286Gly | Heterozygous | AR | 0.0001273 | - | 24 |
|  |  | *PRKDC* | ENST00000338368 | c.4616C>T | p.Thr1539Met | Heterozygous | AR | 0.00002036 | - | 0 |
|  |  | *CARMIL2* | ENST00000334583 | c.3842G>A | p.Ser1281Asn | Heterozygous | AR; AD | 0.000008259 | - | 16.3 |
|  |  | *SERPING1* | ENST00000278407 | c.286A>C | p.Thr96Pro | Heterozygous | AR; AD | 0 | - | 0 |
| P32 | Somalian | *IL6ST* | ENST00000381298 | c.1855G>T | p.Glu619* | Heterozygous | AR; AD | 0 | 0 | 53 |
|  |  |  |  | c.1841C>A | p.Ala614Asp | Heterozygous |  | 0 | 0 | 24.4 |
|  |  |  |  | c.1843C>A | p.Gln615Lys | Heterozygous |  | 0 | 0 | 22.7 |
|  |  | *TCF3* | ENST00000262965 | c.23C>T | p.Ala8Val | Heterozygous | AR; AD | 0.00008529 | 0.00005028 | 26.7 |
|  |  | *NKX2-5* | ENST00000329198 | c.124G>C | p.Ala42Pro | Heterozygous | AD | 0.0002282 | 0.002318 | 20.4 |
|  |  |  |  | c.169G>C | p.Ala57Pro | Heterozygous |  | 0 | 0 | 19.7 |
|  |  | *ITGB2* | ENST00000302347 | c.1501A>G | p.Asn501Asp | Heterozygous | AR | 0.000003984 | 0 | 23.7 |
|  |  | *POLA1* | ENST00000379059 | c.994G>A | p.Val332Ile | Heterozygous | XR | 0.0002394 | 0.002428 | 16 |
|  |  | *SLCO2A1* | ENST00000310926 | c.1291C>G | p.Pro431Ala | Heterozygous | AR; AD | 0.00008854 | 0.0002404 | 0.2 |
|  |  | *SERPING1* | ENST00000278407 | c.304C>A | p.Pro102Thr | Heterozygous | AR; AD | 0 | 0 | 1.1 |
|  |  | *IFNGR1* | ENST00000367739 | c.431A>C | p.Asp144Ala | Heterozygous | AR; AD | 0 | 0 | 18.8 |
|  |  | *CFP* | ENST00000247153 | c.913A>G | p.Ile305Val | Heterozygous | XR | 0.00000558 | 0 | 8.5 |
| P33 | Unknown | *TBK* | ENST00000331710 | c.965A>G | p.His322Arg | Heterozygous | AD | 0.000008356 | - | 23.6 |
|  |  | *ZNF341* | ENST00000375200 | c.334C>T | p.Arg112Cys | Heterozygous | AR | 0.00001777 | - | 29.7 |
|  |  | *ARHGEF1* | ENST00000337665 | c.2525C>T | p.Ala842Val | Heterozygous | AR | 0 | - | 24.7 |
|  |  | *PHRF1* | ENST00000264555 | c.3056C>A | p.Ser1019Tyr | Heterozygous |  | 0.000007268 | - | 22.9 |
|  |  | *CD3E* | ENST00000361763 | c.448T>C | p.Tyr150His | Heterozygous | AR | 0.00001768 | - | 20.7 |
| P34 | Swedish | *VPS13B* | ENST00000358544 | c.2596G>A | p.Val866Ile | Heterozygous | AR | 0.0006151 | 0.002028 | 25 |
|  |  | *AK2* | ENST00000354858 | c.602A>T | p.Tyr201Phe | Heterozygous | AR | 0.0002909 | 0.0002367 | 24.9 |
|  |  |  |  | c.614G>A | p.Gly205Glu | Heterozygous |  | 0.0000502 | 0 | 27.9 |
|  |  | *COL7A1* | ENST00000328333 | c.5820G>A | splice variant | Heterozygous | AR; AD | 0.0000957 | 0 | 18.5 |
|  |  | *MRE11* | ENST00000323929 | c.1138C>T | p.Arg380Cys | Heterozygous | AR | 0.00005174 | 0.0002296 | 30 |
|  |  | *ERCC6L2* | ENST00000288985 | c.1457del | p.Ile486ThrfsX36 | Heterozygous | AR | 0.0004783 | 0.00003827 | 33 |
|  |  | *WAS* | ENST00000376701 | c.1324A>C | p.Ile442Leu | Heterozygous | XR | 0 | 0 | 23.6 |
|  |  | *DOCK11* | ENST00000276202 | c.2563-8A>C | splice variant | Heterozygous | XR | 0.0002696 | 0.0005986 | 10 |
| P35 | Armenian | *RAG1* | ENST00000299440 | c.1060C>A | p.Leu354Met | Heterozygous | AR | 0.0000199 | - | 24.1 |
|  |  | *VPS13B* | ENST00000358544 | c.7787C>T | p.Ser2596Phe | Heterozygous | AR | 0.0007268 | - | 22.7 |
|  |  |  |  | c.3811A>T | p.Thr1271Ser | Heterozygous |  | 0.0007221 | - | 23.6 |
|  |  | *MAP3K14* | NM_003954.5 | c.2780C>A | p.Pro927His | Heterozygous |  | 0.0001121 | - | 16.1 |
|  |  |  |  | c.1627G>A | p.Asp543Asn | Heterozygous |  | 0.0001147 | - | 16.2 |
|  |  | *C8B* | ENST00000371237 | c.362G>C | p.Arg121Pro | Heterozygous | AR | 0.000007958 | - | 21.3 |
|  |  | *MASP1* | ENST00000337774 | c.1556-4C>A | splice variant | Heterozygous | AR | 0 | - | 14.6 |
|  |  | *AEBP1* | ENST00000223357 | c.2864C>G | p.Ala955Gly | Heterozygous | AR | 0.000003996 | - | 24.5 |
|  |  | *IFNAR1* | ENST00000270139 | c.308G>A | p.Arg103His | Heterozygous | AR | 0.00006016 | - | 26.2 |
|  |  | *ADAM17* | ENST00000497473 | c.2047T>C | p.Phe683Leu | Heterozygous | AR | 0.00001598 | - | 23 |
|  |  | *IFIH1* | ENST00000263642 | c.2962G>T | p.Val988Leu | Heterozygous | AR; AD | 0.0004128 | - | 25.4 |
|  |  | *XIAP* | ENST00000371199 | c.878-5C>T | splice variant | Heterozygous | XR | 0.0002045 | - | 4 |
|  |  | *CD46* | ENST00000360212 | c.1006G>A | p.Gly336Ser | Heterozygous | AR; AD | 0.00002785 | - | 15.8 |
|  |  | *PDCD1* | ENST00000334409 | c.512T>C | p.Val171Ala | Heterozygous |  | 0 | - | 7 |
| P36 | Filipino | *G6PD* | ENST00000393562 | c.1450C>T | p.Arg484Cys | Heterozygous | XR | 0.0001422 | 0.0007398 | 29.2 |
|  |  | *NOTCH1* | ENST00000277541 | c.3034G>A | p.Gly1012Ser | Heterozygous | AD | 0.00002334 | 0 | 27.4 |
|  |  | *TTC7A* | ENST00000319190 | c.2515G>A | p.Ala839Thr | Heterozygous | AR | 0.00001992 | 0.00006942 | 27.2 |
|  |  | *GUCY2C* | ENST00000261170 | c.3215A>G | p.Tyr1072Cys | Heterozygous | AR; AD | 0.0004074 | 0.005754 | 27.6 |
|  |  | *EPG5* | ENST00000282041 | c.866A>G | p.Glu289Gly | Heterozygous | AR | 0.000004008 | 0.0000713 | 26.5 |
|  |  | *FCN3* | ENST00000270879 | c.395T>G | p.Val132Gly | Heterozygous | AR | 0 | 0 | 23.7 |
|  |  | *SKIC2* | ENST00000375394 | c.32C>T | p.Pro11Leu | Heterozygous | AR | 0 | 0 | 24.7 |
|  |  | *MRE11* | ENST00000323929 | c.106A>G | p.Thr36Ala | Heterozygous | AR | 0.000007958 | 0 | 21.6 |
|  |  | *CARD14* | ENST00000570421 | c.1829G>A | p.Arg610His | Heterozygous | AD | 0.0001719 | 0.001255 | 25.7 |
|  |  | *RTEL1* | ENST00000370018 | c.2987C>A | p.Pro996His | Heterozygous | AR; AD | 0.000219 | 0.002846 | 11 |
|  |  | *RC3H1* | ENST00000258349 | c.1117A>G | p.Thr373Ala | Heterozygous |  | 0.00006718 | 0.0008319 | 23.2 |
|  |  | *DOCK2* | ENST00000256935 | c.5153C>T | p.Ser1718Leu | Heterozygous | AR | 0.000124 | 0.001873 | 22 |
|  |  |  |  | c.979+6G>A | splice variant | Heterozygous |  | 0.0005651 | 0.007244 | 0.1 |
|  |  | *DDX58* | ENST00000379883 | c.1991G>A | p.Arg664His | Heterozygous | AD | 0.00005007 | 0.0004888 | 23.6 |
|  |  |  |  | c.2014+8T>C | splice variant | Heterozygous |  | 0.0001628 | 0.0004924 | 7.7 |
|  |  | *MYO5A* | ENST00000399231 | c.5326A>G | p.Thr1776Ala | Heterozygous | AR | 0.0001211 | 0.0006417 | 22.1 |
|  |  | *MEFV* | ENST00000219596 | c.547C>A | p.Pro183Thr | Heterozygous | AR; AD | 0.0001391 | 0.002567 | 0 |
|  |  | *SENP3* | ENST00000321337 | c.713C>G | p.Ser238Trp | Heterozygous |  | 0 | 0 | 29.6 |
|  |  |  |  | c.715+4A>T | splice variant | Heterozygous |  | 0 | 0 | 17.2 |
|  |  | *PLEKHG6* | ENST00000011684 | c.1834G>A | p.Ala612Thr | Heterozygous | AD | 0.0001344 | 0.001804 | 24.7 |
|  |  | *LIG1* | ENST00000263274 | c.2086G>A | p.Ala696Thr | Heterozygous | AR | 0.00003537 | 0 | 23.9 |
|  |  | *NEIL3* | ENST00000264596 | c.595C>G | p.Leu199Val | Heterozygous |  | 0.0009482 | 0.01255 | 25 |
|  |  | *HYOU1* | ENST00000404233 | c.1111G>C | p.Glu371Gln | Heterozygous | AR | 0.000283 | 0.002565 | 21.3 |
|  |  | *MVD* | ENST00000301012 | c.985G>A | p.Gly329Ser | Heterozygous | AR; AD | 0.0002061 | 0.002059 | 10.3 |
|  |  | *CORO1A* | ENST00000219150 | c.347T>C | p.Leu116Pro | Heterozygous | AR | 0.0001828 | 0 | 21.6 |
|  |  | *NOD2* | ENST00000300589 | c.392C>A | p.Ala131Asp | Heterozygous | AD | 0 | 0 | 17.3 |
|  |  | *CASP10* | ENST00000286879 | c.314T>C | p.Leu105Pro | Heterozygous | AD | 0.00003955 | 0.0002781 | 0.2 |
|  |  | *PMS2* | ENST00000265849 | c.1354G>C | p.Gly452Arg | Heterozygous | AR | 0.00001988 | 0.0003466 | 0.9 |
| P37 | Albanian | *MSH6* | ENST00000234420 | c.3260C>A | p.Pro1087His | Heterozygous | AR; AD | 0.0001237 | 0.00009055 | 24.8 |
|  |  | *TNFRSF13B* | ENST00000261652 | c.605G>A | p.Arg202His | Heterozygous | AR; AD | 0.0007751 | 0.001366 | 13 |
|  |  | *ELANE* | ENST00000263621 | c.511G>A | p.Glu171Lys | Heterozygous | AD | 0.00002876 | 0.00009318 | 24.3 |
|  |  | *EPG5* | ENST00000282041 | c.5472C>A | p.Tyr1824* | Heterozygous | AR | 0 | 0 | 39 |
|  |  | *NBAS* | ENST00000281513 | c.4357T>G | p.Cys1453Gly | Heterozygous | AR | 0.00002788 | 0.00009718 | 15 |
|  |  |  |  | c.4351C>G | p.Gln1451Glu | Heterozygous |  | 0.00002791 | 0.00009735 | 16.6 |
|  |  | *POLD1* | ENST00000440232 | c.2803G>A | p.Ala935Thr | Heterozygous | AD | 0.00003743 | 0.000101 | 27.2 |
|  |  | *AP4B1* | ENST00000256658 | c.175A>G | p.Thr59Ala | Heterozygous | AR | 0.00009544 | 0.0003923 | 22.9 |
|  |  | *RNASEH2A* | ENST00000221486 | c.662A>G | p.Lys221Arg | Heterozygous | AR | 0.0006119 | 0.0006647 | 4.7 |
| P38 | Albanian | *MSH6* | ENST00000234420 | c.3260C>A | p.Pro1087His | Heterozygous | AR; AD | 0.0001237 | 0.00009055 | 24.8 |
|  |  | *ELANE* | ENST00000263621 | c.511G>A | p.Glu171Lys | Heterozygous | AD | 0.00002876 | 0.00009318 | 24.3 |
|  |  | *NBAS* | ENST00000281513 | c.4357T>G | p.Cys1453Gly | Heterozygous | AR | 0.00002788 | 0.00009718 | 15 |
|  |  |  |  | c.4351C>G | p.Gln1451Glu | Heterozygous |  | 0.00002791 | 0.00009735 | 16.6 |
|  |  | *TRAF3* | ENST00000560371 | c.-18+4A>C | splice variant | Heterozygous |  | 0 | 0 | 10.3 |
|  |  | *TNFRSF13B* | ENST00000261652 | c.418G>A | p.Glu140Lys | Heterozygous | AR; AD | 0.00002386 | 0 | 22.8 |
|  |  | *AP4B1* | ENST00000256658 | c.175A>G | p.Thr59Ala | Heterozygous | AR | 0.00009544 | 0.0003923 | 22.9 |
|  |  | *RNASEH2A* | ENST00000221486 | c.662A>G | p.Lys221Arg | Heterozygous | AR | 0.0006119 | 0.0006647 | 4.7 |
|  |  | *TNFRSF11A* | ENST0000586569 | c.932C>T | p.Thr311Ile | Heterozygous | AR; AD | 0.00002784 | 0.00006452 | 0.4 |
|  |  | *FERMT1* | ENST00000217289 | c.524G>C | p.Gly175Ala | Heterozygous | AR | 0.00003182 | 0.00009685 | 14.1 |
|  |  | *C5* | ENST00000223642 | c.4505-7G>A | splice variant | Heterozygous | AR | 0.0001523 | 0.0002425 | 12 |

Abbreviations: AR, autosomal recessive; AD, autosomal dominant; XR, X-linked recessive; XD, X-linked dominant

* Data retrieved with VEP

^a^ Ensembl reference sequences; ^b^ gene inheritance model predicted by OMIM; ^c^ minor allele frequency according to gnomAD database*; ^d^ CADD pathogenicity score*

**Supplementary figure legends**

**Supplementary Figure 1 Clinical features of our critical COVID-19 cohort. (A)** Plot shows the BMI and time spent in the ICU for all cohort patients. BMI had a significant positive correlation with required time receiving critical care when patients with autoantibodies were not included (black). Significant correlation was not observed when autoantibody patients were included (red). **(B)** The proportion of patients who answered affirmatively when asked if they had the most commonly present COVID-19 symptoms at three different time-points related to hospital admission for COVID-19 treatment.

**Supplementary Figure 2 Patient T cell counts during convalescence (A)** CD4+ and CD8+ counts from whole blood were taken from the patients and healthy controls. A significant difference between CD4+ central memory cells in patients and healthy controls was identified; otherwise no significant difference was observed in cell counts between the two groups. **(B)** T-blasts per ul were measured after exposure to SARS-CoV-2 or Influenza virus in patients and healthy controls. Whiskers extend from the box to the most extreme point still within 1.5 times the interquartile range from the box; points beyond this are shown and deemed outliers.

**Supplementary Figure 3 Visualisation of the CNVs in 1 Mb surrounding each autosomal gene of interest. (A:AA)** In alphabetical order, any copy number variant in a minimum of 1 Mb surrounding the gene is shown. No CNVs overlapping gene exons were predicted.

**Supplementary Figure 4 PCA of ancestry analysis. (A)** Patient genomes were merged with the 1kGP to give an overlay in the PCA. Patient points are colored according to the pathway of any variants discovered in their genomes (green for the type I IFN signaling pathway, yellow for the TLR7 and TLR9 pathways, blue for the TLR3 pathway), or red for patients with detectable autoantibodies.

**Supplementary Figure 5 Odds ratios of Type I IFN genes aligned to odds ratios of handedness-associated genes with similar constraint metrics for (A)** all autosomal genes included in the study; **(B)** genes in the type I IFN signaling pathway and the cumulative variants of all genes in the type I IFN signaling pathway. For the handedness-associated genes, this is the cumulative variants of all the genes used as controls for the type I IFN signaling pathway genes.

**Supplementary Figure 6 Sensitivity analysis of obesity in the Firth’s bias-reduced regression model using simulated BMI data for the 1000 Genomes Project.** **(A)** Histogram of BMI distribution in each of the simulations performed for the 1000 Genomes project. **(B)** Distance of the OR without BMI as a parameter from the mean of the ORs with simulated BMI included as a covariate.

**Supplementary Figure 7 Immunological assays performed on patient cells. (A)** pDC counts from whole blood were taken from the patients and healthy controls. No significant difference was observed in cell counts between the two groups. **(B)** Fold change of pSTAT1 and pSTAT2 CD4^+^ T-cells was correlated when these were stimulated with IFN-α, in both patient and healthy control samples. **(C)** Correlation was calculated for change in pSTAT1, pSTAT2, and interferon-stimulated genes MX1, IRF7, and IFIT1, between CD4^+^ T-cells and CD8^+^ T-cells, and visualised as a matrix.
